# Flow Cytometry Based Detection and Isolation of *Plasmodium falciparum* Liver Stages *In Vitro*

**DOI:** 10.1371/journal.pone.0129623

**Published:** 2015-06-12

**Authors:** Peter C. Dumoulin, Stefanie A. Trop, Jinxia Ma, Hao Zhang, Matthew A. Sherman, Jelena Levitskaya

**Affiliations:** 1 W. Harry Feinstone Department of Molecular Microbiology and Immunology, Bloomberg School of Public Health, Johns Hopkins University, Baltimore, MD, 21205, United States of America; 2 Triangle Research Labs, 6 Davis Drive, Durham, NC, 27709, United States of America; INSERM, FRANCE

## Abstract

Malaria, the disease caused by *Plasmodium* parasites, remains a major global health burden. The liver stage of *Plasmodium falciparum* infection is a leading target for immunological and pharmacological interventions. Therefore, novel approaches providing specific detection and isolation of live *P*. *falciparum* exoerythrocytic forms (EEFs) are warranted. Utilizing a recently generated parasite strain expressing green fluorescent protein (GFP) we established a method which, allows for detection and isolation of developing live *P*. *falciparum* liver stages by flow cytometry. Using this technique we compared the susceptibility of five immortalized human hepatocyte cell lines and primary hepatocyte cultures from three donors to infection by *P*. *falciparum* sporozoites. Here, we show that EEFs can be detected and isolated from *in vitro* infected cultures of the HC-04 cell line and primary human hepatocytes. We confirmed the presence of developing parasites in sorted live human hepatocytes and characterized their morphology by fluorescence microscopy. Finally, we validated the practical applications of our approach by re-examining the importance of host ligand CD81 for hepatocyte infection by *P*. *falciparum* sporozoites *in vitro* and assessment of the inhibitory activity of anti-sporozoite antibodies. This methodology provides us with the tools to study both, the basic biology of the *P*. *falciparum* liver stage and the effects of host-derived factors on the development of *P*. *falciparum* EEFs.

## Introduction

Infection with *Plasmodium* parasites, the causative agent of malaria, remains a major public health problem. In 2012 an estimated 207 million new cases of malaria occurred resulting in an estimated 627,000 deaths, primarily in sub-Saharan Africa [1]. Of the five currently known human malaria parasites, *Plasmodium falciparum* causes the highest rates of complications and mortality [1]. The *Plasmodium* life cycle in humans consists of two phases: the clinically silent liver stage, or exoerythrocytic form (EEF), and the erythrocytic stage [2, 3]. The latter is routinely studied both *in vitro* [4] using red blood cell cultures and *ex vivo* using patient-derived infected blood [5–7].

Direct access to *in vivo* infected human hepatocytes is untenable due to ethical and logistic constraints. Consequently, studies of the liver stage of *Plasmodium* infection have relied mainly on the use of rodent parasites both *in vivo* and *in vitro* [8, 9]. The rodent parasites *Plasmodium berghei* and *Plasmodium yoelii* complete full development in the hepatocyte in less than three days after infection and can fully develop in human hepatocellular carcinoma cell lines *in vitro* [10, 11]. However, the human parasite *P*. *falciparum* requires at least 144 hours for full EEF development in the liver and has a limited ability to infect human hepatocellular carcinoma cell lines [9].

Multiple experimental models utilizing primary human hepatocytes for *P*. *falciparum* EEF development have been reported. Infection of primary hepatocytes *in vitro* by *P*. *falciparum* was first described almost thirty years ago [12]. Recent work using micropatterned primary hepatocytes surrounded by stromal cells has allowed for both complete development of *P*. *falciparum* EEFs and possibly generation of *P*. *vivax* hypnozoites *in vitro* [13]. The first mouse models relying on the engraftment of human hepatocytes into immune-compromised animals capable of generating mature EEFs were reported more than two decades ago [14] and were further used to obtain isolated infected cells from fixed frozen liver tissues through micro-dissection [15]. Complete development of *P*. *falciparum* liver stages and liver-to-blood transmission was later demonstrated *in vivo* in immune-compromised and fumarylacetoacetate hydrolase-deficient animals backcrossed with NOD mice [16]. Recently, SCID mice with chimeric human livers were used to show the protective effect of parasite antigen-specific human monoclonal antibodies derived from RTS,S vaccine recipients [17].

The *in vivo* and *in vitro* methods described above demonstrated the generation of *P*. *falciparum* merozoites capable of infecting red blood cells. However, the technical complexity and high associated costs restrict the widespread use of these methodologies for routine studies on *P*. *falciparum* liver stages. Additionally, these methods rely on immunofluorescence or quantification of total parasite biomass and are unable to isolate live, individual *P*.*falciparum* EEFs. Therefore, a technically reproducible and cost-effective experimental system for *in vitro* monitoring and purification of *P*. *falciparum* EEFs is still needed.

Mouse models of the liver stage of malaria infection suggest a role for both CD8^+^ T cells and sporozoite antigen-specific antibodies in sterilizing immunity [18]. However, understanding the contributions of humoral and cell mediated immune responses directed against *P*. *falciparum* EEFs during the natural course of infection [19, 20] or induced upon vaccination [21, 22] requires a robust *in vitro* system.

Two modes of interaction between sporozoites and host hepatocytes are currently described *in vitro* [23, 24] and *in vivo* [25, 26]: (i) breaching of the host cell plasma membrane followed by intracellular movement and subsequent exit, referred to as traversal, and (ii) productive invasion and parasitophorous vacuole formation within hepatocytes. The influence of traversed cells on infection and parasite biology are largely unknown. Thus, an optimal experimental *in vitro* system recapitulating the liver stage of *P*. *falciparum* should allow for specific identification and isolation of traversed from non-traversed and infected from non-infected cells. In experimental models of *Plasmodium* infection *in vitro* non-traversed and non-infected populations are similarly exposed to a plethora of biological factors from the salivary glands of infected mosquitoes. Thus, these populations of hepatocytes serve as the most accurate control to study the immunology and developmental biology of *P*. *falciparum* liver stage infection *in vitro*. However, *in vivo*, sporozoites are deposited into the host dermis and the direct impact of salivary gland content on hepatocytes is likely to be insignificant [27].

Here we report a flow cytometry based *in vitro* system to monitor *P*. *falciparum* liver stages that permits (i) detection and isolation of *P*. *falciparum* EEFs, (ii) evaluation of host factors on the establishment of an exoerythrocytic infection, and (iii) efficacy assessment of antibodies blocking sporozoite motility.

## Materials and Methods

### Human hepatocyte culture

HC-04 [28] was obtained from the ATCC (Manassas, VA, USA). HepG2 and HepG2-CD81 [29] were kindly provided by Dr. I. Coppens (Johns Hopkins Bloomberg School of Public Health). These cell lines were maintained in IMDM supplemented with 2.5% FCS, 100 IU/ml penicillin, 100 μg/ml streptomycin and 2 mM L-glutamine. THLE-2 and THLE-3 cell lines [30] were kindly provided by Dr. C. Harris (NCI, Center for Cancer Research) and maintained using BEGM BulletKit medium (Lonza, Walkersville, MD) supplemented with 5 ng/ml EGF, 70 ng/ml phosphoethanolamine and 10% FCS. All cell lines were routinely tested for the presence of mycoplasma [31].

Cryopreserved human primary hepatocytes, hepatocyte thawing, plating and maintenance medium were obtained from Triangle Research Labs (TRL, North Carolina). Hepatocytes were thawed in a 37°C water bath for 2 minutes in thawing medium, spun at 100x*g* for 8 minutes and plated on collagen-coated wells or coverslips in plating medium. Plating medium was replaced with maintenance medium supplemented with 10% FCS 6 hours after plating and replaced every 24 hours.

### Infection with *P*. *falciparum* sporozoites

Prior to addition of sporozoites, HC-04, HepG2 and HepG2-CD81 were seeded at densities of 400,000, 200,000 or 100,000 cells/well in 12, 24 or 48 well plates respectively, to achieve confluence. THLE-2 and THLE-3 were seeded at 200,000, 100,000 or 50,000 cells/well in 12, 24 or 48 well plates to achieve confluence. Primary hepatocytes were seeded at 300,000 hepatocytes per well in a 24 well plate 48 hours prior to addition of sporozoites. Sporozoite to hepatocyte ratios are indicated for each experiment in relevant figure legends. *Plasmodium falciparum* 3D7HT-GFP [32] sporozoites were isolated and purified by density gradient as described previously [33]. Sporozoites were added to hepatocytes in IMDM with 2.5% FCS supplemented with penicillin, streptomycin and L-glutamine, centrifuged at 380x*g* for 5 minutes without break and then incubated at 37°C for 4–6 hours. After incubation, cells were washed with PBS and maintained in complete growth medium supplemented with Fungizone (1.25ug/ml, Gibco, Grand Island, NY). To prevent overgrowth of HC-04, HepG2, and HepG2-CD81 cells, each well was individually processed at 24 hours postinfection (pi), split at a 1:2 ratio and re-seeded separately into a new plate. Medium was changed daily. Fungizone treatment was terminated at 48 hours pi.

### Hepatocyte traversal

Hepatocyte traversal was performed as previously described [23, 24]. At the time of sporozoite addition or mock infection medium was supplemented with 0.2 mg/ml of 10,000 MW dextran-tetramethylrhodamine (Life Technologies, Grand Island, NY). After incubation at 37°C for 6 hours cells were washed three times in PBS, trypsinized and re-suspended in PBS/0.1% BSA. Dextran retention was detected using FACSCalibur and CellQuest acquisition software (Becton Dickinson, New Jersey, USA). Data analyses were performed using FlowJo software (TreeStar Inc., Ashland Oregon, USA).

### Detection of infected hepatocytes by flow cytometry

Hepatocytes from individual wells were washed three times in PBS, trypsinized, re-suspended in PBS/0.1% BSA and analyzed using a Becton Dickinson LSR II or FACSCalibur (Becton Dickinson, New Jersey, USA). Dead cells were excluded by propidium iodide (PI) staining. Data analyses were performed using FlowJo software (TreeStar Inc., Ashland Oregon, USA). GFP-positive PI-negative events were identified using a ratio of the FL1 (530/30) and FL2 (585/42) fluorescence channels. For quantification of infected cell populations, percentages of GFP^+^ cells as well as geometric mean fluorescent intensity (geoMFI) of the gated GFP positive viable (PI-negative) population are indicated.

### Quantification of infection by real-time PCR

Hepatocytes were lysed using the Power SYBR Green Cells-to-C_T_ kit (Ambion, Grand Island, NY) at the density of 2000 cells/μl. The lysis reaction was stopped with a proportional volume of 10x Stop Solution. Reverse transcription was performed using 22.5 μl of cell lysate in a total volume of 50 μl, and 4 μl of the resulting cDNA were used as template per PCR reaction. PCR amplification used the following primer pairs: Human GAPDH 5′-gcaaattccatggcaccgt-3′/5′-tcgccccacttgattttgg-3′ *P*. *falciparum* 18S 5′-tcagataccgtcgtaatctta-3′/5'-aactttctcgcttgcgcgaa-3′ [34]. Each sample was amplified in triplicate using an Applied Biosystems StepOnePlus and SYBR Green for detection. Thermal cycling proceeded for 2 minutes at 50°C, 10 minutes at 95°C followed by 40 cycles of 15 seconds at 95°C and 1 minute at 60°C. *Pf*18S copy numbers were calculated using a standard curve generated from serial dilution of a plasmid template containing the *Pf*18S gene. Samples were normalized to their own GAPDH C_T_ values.

### Immunofluorescence of GFP-positive HC-04 and primary hepatocytes

GFP-positive HC-04 cells were sorted 96 hours pi directly into 4% paraformaldehyde using a Beckman Coulter MoFlo Cell Sorter (Johns Hopkins Bloomberg School of Public Health, Flow Cytometry and Cell Sorting Core Facility). Fixed cells were cytospun at 1500 rpm for 5 minutes onto cytoslides (Thermo Shandon, Astmoor, UK). Specimens were blocked and permeabilized in PBS containing 10% goat serum, 1% BSA, and 0.1% Triton X-100, and then stained with anti-*Pf*Hsp70 (cl. 4C9, F. Zavala, JHU) [35] followed by anti-mouse IgG-Alexa Fluor 594 (Molecular Probes, Grand Island, NY). Slides were mounted with ProLong Gold Antifade containing DAPI (Molecular Probes, Eugene, OR) and viewed on a widefield Nikon 90i microscope. Data were acquired and processed with Volocity software (Perkin Elmer, Waltham, MA).

Primary hepatocytes seeded on collagen-coated glass coverslips were infected, fixed and stained with an anti-*Pf*Hsp70 antibody at 96 hours pi as described above. Quantifications of parasite size and distance from host nucleus were performed in Volocity software, using the *Pf*Hsp70-specific fluorescence signal as margins for surface area and longest axis calculations. Minimum distance was calculated from the edge of *Pf*Hsp70 to the edge of the nearest hepatocyte nucleus.

### CD81 detection and neutralization

Single cell suspensions of hepatocytes were incubated for 20 minutes on ice with either anti-CD81 (clone JS-81, BD Pharmingen, San Diego, CA) or IgG1 isotype control antibody, each conjugated to APC. Azide-free anti-CD81 mouse monoclonal antibody (cl.1D6) (Abcam, Cambridge, MA) or a functional grade IgG1 isotype control antibody (eBioscience, San Diego, CA) were used in CD81 blocking experiments at a final concentration of 10 μg/ml. Antibodies were either added 2 hours prior to infection and removed, added concurrently with sporozoites, or added at 6 hours pi.

### Evaluation of efficacy of mouse serum containing humanized anti-CSP mAb 2A10

Mouse serum containing humanized CSP-specific monoclonal antibody 2A10 [36–38] (h2A10) was kindly provided by Dr. Gary Ketner and Dr. Cailin Deal (Johns Hopkins Bloomberg School of Public Health) [39]. Briefly, sera samples were collected from a single mouse prior to and 11 weeks after intra-muscular administration of 1x10^11^ genome copies of AAV-expressing humanized 2A10 (anti-CSP). Concentration of human IgG in total immune serum was 1.93 mg/ml. Serum were added at the same time as sporozoites.

### Statistical analysis

Comparisons of multiple groups were performed with a one way ANOVA. A post-hoc Tukey’s test was used for comparisons between individual groups. Adjusted p-values are shown where relevant (*p<0.05, **p<0.01, ***p<0.001). There was considerable variability in infectivity between sporozoite preparations; consequently, relevant comparisons are made only between sporozoites from the same preparations or are normalized where relevant.

## Results

### Immortalized human hepatocyte cell lines are permissive for traversal by *P*. *falciparum* sporozoites

Traversal of hepatocytes by *Plasmodium* sporozoites has been previously documented *in vitro* [23, 24] and *in vivo* [25, 26]. The *in vitro* traversed cell population was identified by flow cytometry by its ability to retain fluorescent dextran during migration of sporozoites through the host cell [24]. Gating strategy for flow cytometry-based detection of hepatocytes traversed with *P*. *falciparum* 3D7HT-GFP sporozoites is shown (S1A Fig). In search of cell lines that allow for efficient detection of traversal and invasion of *P*. *falciparum* sporozoites, we turned to immortalized cell lines derived from human hepatocytes that included THLE-2 and THLE-3 [30], HepG2, HepG2-CD81 [29] and HC-04. HC-04 is the only cell line previously reported to support development of *P*. *falciparum* liver stages [13, 28].

**Fig 1:**
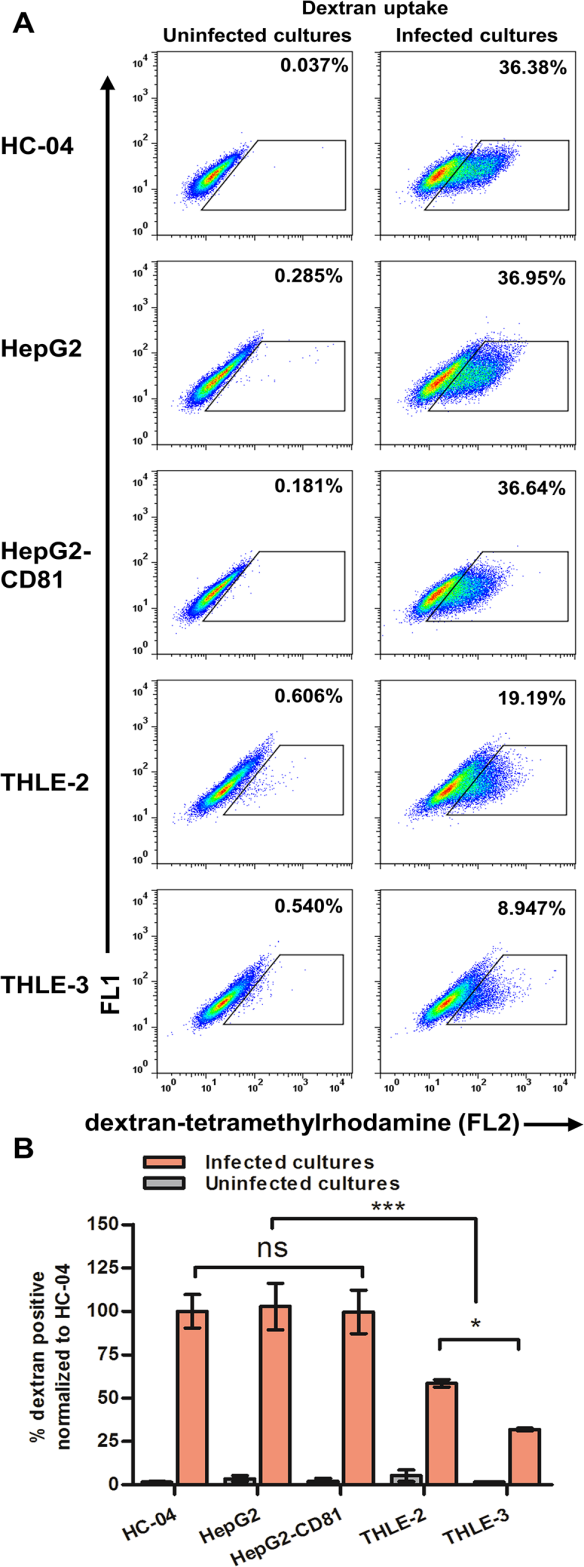
Human hepatocyte cell lines can be traversed by *P*. *falciparum* sporozoites. Traversal was assessed by flow cytometry as an ability of cells to uptake and retain fluorescent high molecular weight dextran during sporozoite co-incubation for 6 hours. (A) Representative plots for each cell line are shown, 1.5:1 sporozoite-to-hepatocyte ratio was used. Numbers indicate percentage of dextran-positive cells. (B) Percentage of traversed cells normalized to HC-04 obtained from three sporozoite preparations, each experiment was conducted in triplicate. Mean +/- SD shown.

We found that all five cell lines tested had a detectable population of hepatocytes traversed by *P*. *falciparum* 3D7HT-GFP sporozoites (Fig 1A). HC-04, HepG2 and HepG2-CD81 had comparable percentages of cells retaining fluorescent dextran, which was about 2–3 fold higher than in cultures of THLE-2 and THLE-3 cell lines (Fig 1B). We concluded that each of the tested cell lines can be utilized to study traversal by *P*. *falciparum* sporozoites *in vitro*. Traversal was measured at 6 hours after addition of sporozoites. At this time we are unable to detect any GFP-positive hepatocytes by flow cytometry. In agreement with previous studies utilizing rodent parasites [24] we found that fluorescent dextran uptake in human hepatocyte cultures incubated with *P*. *falciparum* sporozoites depends on hepatocyte-to-sporozoite ratio and could be inhibited by preincubation of sporozoites with cytochalasin D, an inhibitor of sporozoite motility (S1B and S1C Fig). Of note, the sporozoite-to-hepatocyte ratios used in our study are significantly higher than those reported previously [24] and, therefore, higher percentages of traversed cells are detected in our experiments. Purification of sporozoites by density the gradient centrifugation method used in our study removes the majority of mosquito salivary gland material that generates a cleaner and more concentrated preparation.

### Human hepatocyte cell lines exhibit a differential ability to support development of *P*. *falciparum* EEFs

Next, to assess the ability of immortalized hepatocyte cell lines to support the development of *P*. *falciparum* 3D7HT-GFP parasites, we used a novel, quantitative, flow cytometry-based analysis of infected cultures. We utilized relative FL1 (530/30) to FL2 (585/42) fluorescence ratios to detect the weak GFP signal of the parasite in relation to background autofluorescence of the host cell while simultaneously excluding dead cells using propidium iodide. To validate our ability to specifically detect developing parasites we quantified parasite 18S expression using real-time PCR and visualized *P*. *falciparum* EEFs by immunofluorescence microscopy following flow cytometry-based sorting of infected cells.

**Fig 2:**
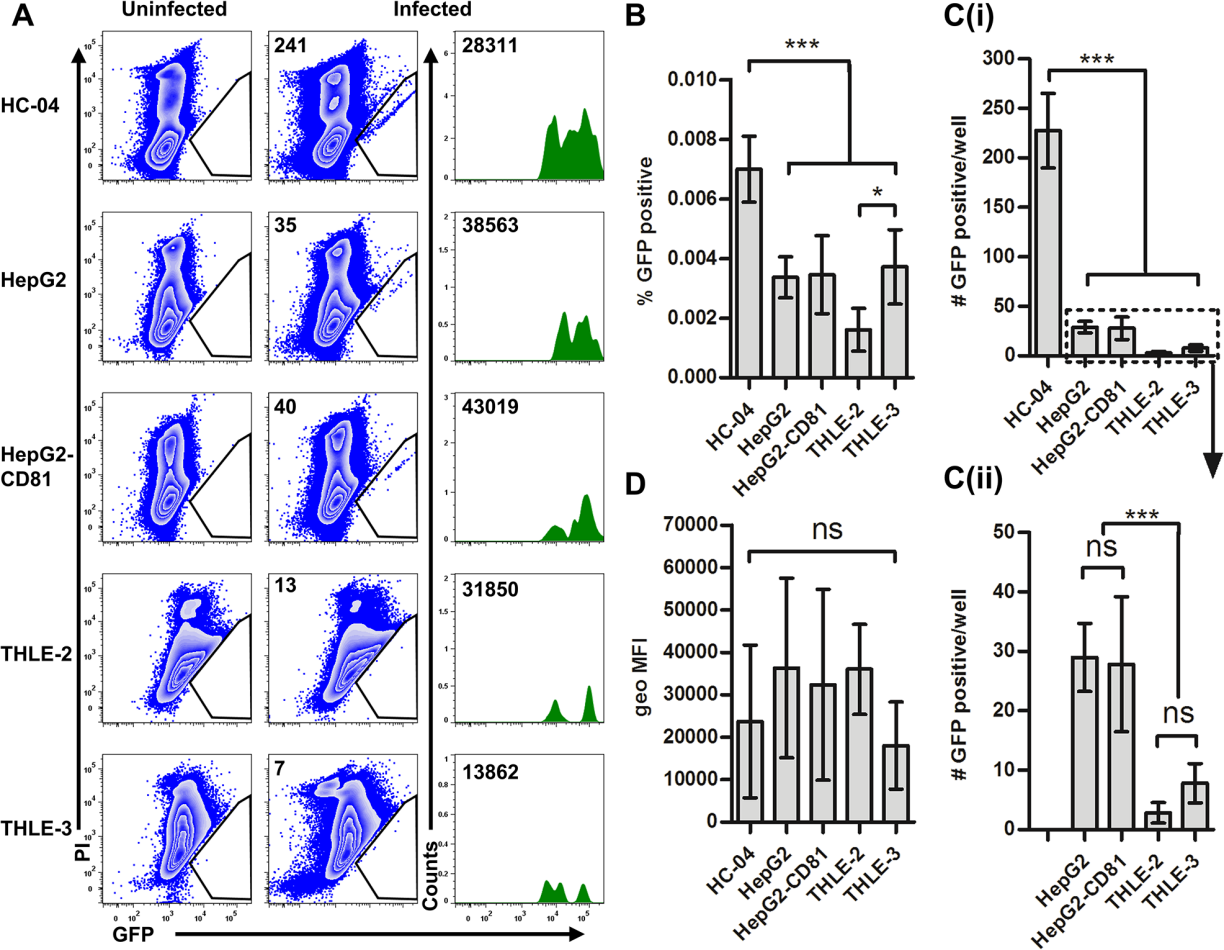
Identification of hepatocytes infected with *P*. *falciparum* 3D7HT-GFP parasites by flow cytometry. Sporozoites were added at a 2.3:1 sporozoite-to-hepatocyte ratio. Infected cells were identified by flow cytometry at 96 hours postinfection as GFP-positive events in PI-negative (viable) cell populations. Uninfected cultures propagated in parallel with infected cultures were used to define specificity of GFP-positive events. A similar number of total events were acquired from infected and uninfected cultures of each cell lines. (A) Data from one representative culture is shown for each cell line. Event number corresponding to the GFP-positive gate (middle panels) and the geometric mean of events in the GFP-positive gate is indicated (right panels). (B) Percentage of GFP-positive PI-negative events. (C) Number of GFP events per well (D) Geometric MFI in FL-1 of GFP-positive PI-negative events. Mean +/- SD shown for all bar graphs (n = 6 per cell line).

Here we show that GFP^+^ events could be detected not only in HC-04, but also in HepG2 and HepG2-CD81, which had been previously described as non-permissive for the development of *P*. *falciparum* EEFs (Fig 2A) [9]. However, the relative frequencies (percentages) of GFP^+^ PI^-^ events were 2.5-fold higher (Fig 2B) and the total number of GFP^+^ events per well were 8-fold higher (Fig 2C) in HC-04 as compared to HepG2 and HepG2-CD81 cells. Both THLE-2 and THLE-3 had significantly fewer total GFP^+^ events than the other cell lines tested (Fig 2C). In contrast, the fluorescence intensity of the GFP^+^ populations, an indication of parasite development, was comparable in all cell lines tested (Fig 2D).

**Fig 3:**
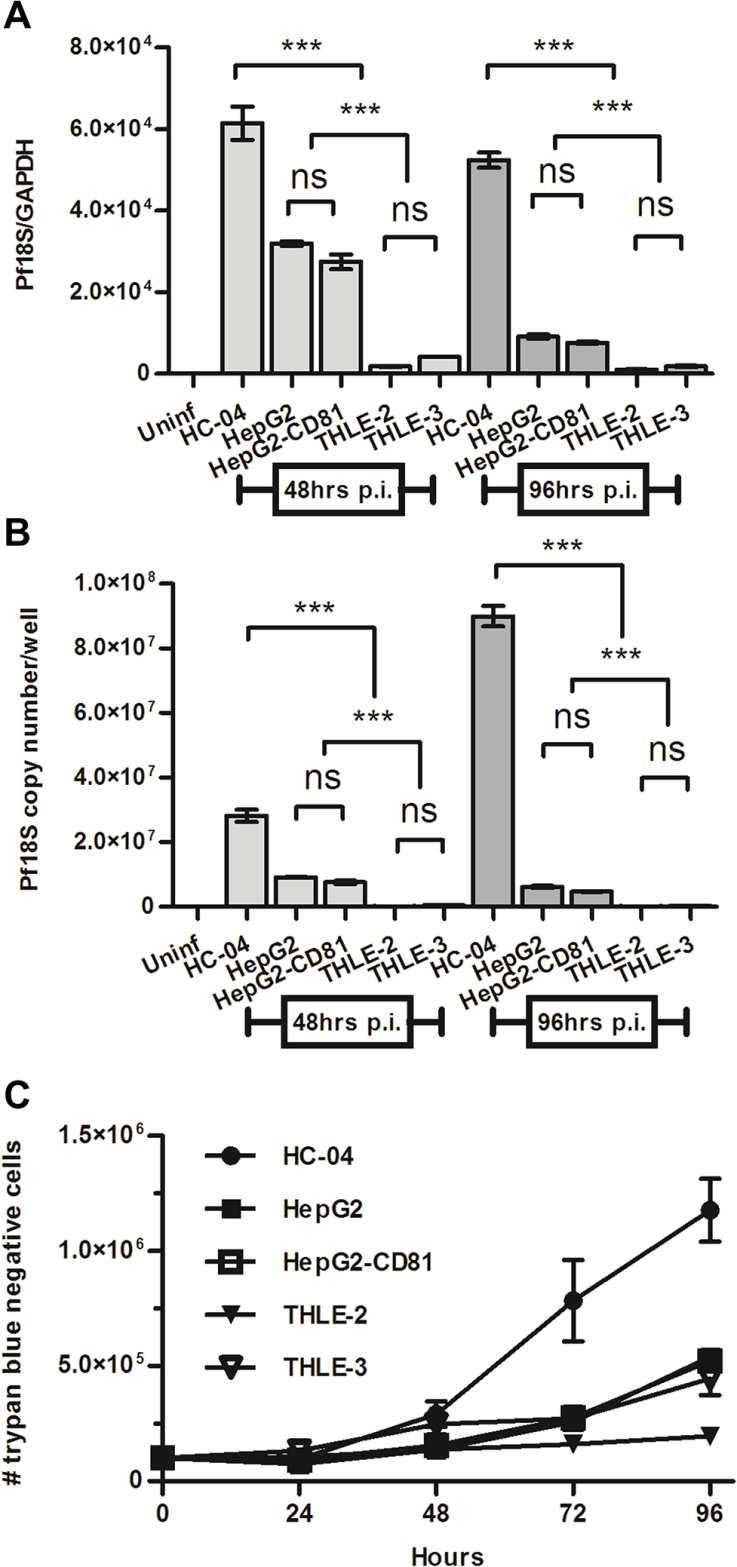
Quantification of parasite-encoded 18S rRNA in cultures of human hepatocytes infected with *P*. *falciparum*. Sporozoites were added at a 2.3:1 sporozoite-to-hepatocyte ratio. Real-time PCR analysis of 18S rRNA expression was done at 48 and 96 hours postinfection. Each culture (n = 3) was analyzed in technical triplicate. (A) 18S copy number normalized using C_T_ values from GAPDH (B) 18S copy number per culture. (C) Growth curve of uninfected hepatocyte cell lines (n = 3 per time point). Mean +/- SD shown for all graphs.

Quantification of *P*. *falciparum* 18S rRNA at two time points postinfection supported our flow cytometry findings (Fig 3). The ratio of parasite 18S copy number relative to a hepatocyte housekeeping gene (GAPDH C_T,_ arbitrary units) was 2-fold higher in HC-04 than in HepG2 and HepG2-CD81, and about 30-fold higher than in THLE-2 and THLE-3 (Fig 3A). In HC-04, a 3-fold increase in the *Pf*18S rRNA copy numbers per well was observed between 48 and 96 hours postinfection (Fig 3B). However, a decrease in the 18S:GAPDH ratio was observed in the same cultures from 48 to 96 hours postinfection (Fig 3A). This can be explained by extensive proliferation of uninfected HC-04 cells (Fig 3C) resulting in the relative drop in percentage of infected hepatocytes.

### *P*. *falciparum*-infected HC-04 hepatocytes can be specifically isolated

Infected hepatocytes from mice have been sorted *ex vivo* using *P*. *yoelii* parasites expressing GFP [40]. We have previously demonstrated that HC-04 cells infected with *P*. *berghei* ANKA-GFP can be isolated by flow cytometry-based cell sorting based on GFP expression *in vitro* [33]. Utilizing a similar approach we performed infection of HC-04 with *P*. *falciparum* 3D7HT-GFP sporozoites and sought to verify that GFP^+^ HC-04 cells contain developing parasites. GFP^+^PI^-^ hepatocytes were sorted and cytospun, followed by immunofluorescence analysis of GFP^+^ cell populations. Though these experimental procedures may alter native host and parasite morphology it was necessary to perform due to a short doubling time (Fig 3C) of uninfected cells leading to extensive three-dimensional growth of HC-04 cultures. Moreover, sorting and visualization of parasites was needed to validate flow cytometry method as an approach for detection and isolation of *P*. *falciparum* infected cells.

**Fig 4:**
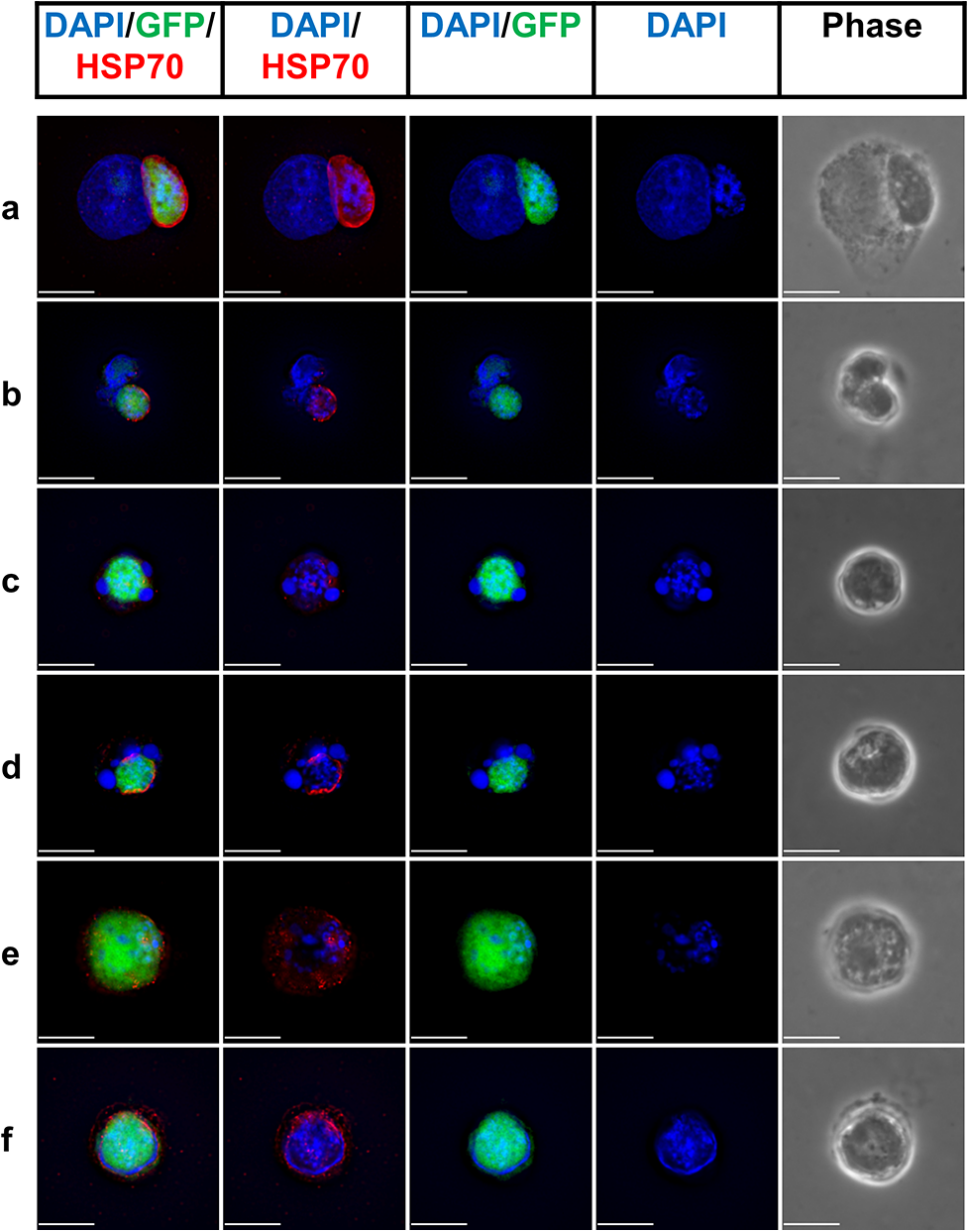
Visualization of parasites in HC-04 cells infected with *P*. *falciparum*. GFP-positive PI-negative events were isolated by flow cytometry-based cell sorting 96 hours postinfection and cytospun. Representative micrographs of EEFs show DNA staining with DAPI, GFP expression and immunofluorescent detection of *Pf*Hsp70 by anti-Hsp70 monoclonal antibody (Blue = DAPI, Green = GFP, Red = *Pf*Hsp70. Scale bars indicate 10 μm).

As shown in Fig 4, GFP^+^ cells isolated at 96 hours pi contained *P*. *falciparum* parasites. We used three fluorescent markers to identify the presence of EEFs: parasite-encoded GFP, DAPI to stain both host and parasite DNA, and a monoclonal antibody against *Pf*Hsp70. We observed three patterns in sorted GFP^+^ cells (Fig 4): (a, b) a GFP-positive, Hsp70 positive parasite located perinuclear and morphologically similar to parasites in primary hepatocytes [12, 13] (c, d) a GFP-positive Hsp70-positive parasite adjacent to a dividing or fragmented host nucleus and (e, f) a GFP-positive Hsp70-positive cell with indistinguishable host DNA. We speculate that the latter group of cells was either devoid of a host nucleus or had a host nucleus containing the parasite. We cannot exclude, however, that some of the hepatocyte cell nuclei were either masked by the parasite or damaged during the cytospin procedure. Since there are no markers available to distinguish each pattern of the parasite by flow cytometry directly, new techniques, such as flow cytometry combined with imaging may become instrumental for further characterization of the parasite patterns in HC-04 cells infected with *P*. *falciparum*. Punctate DNA staining within the parasites is indicative of development. We do not exclusively observe *P*. *falciparum* localized in the nucleus of HC-04 cells as has been previously reported for HepG2 [29], however, we cannot formally exclude that this may occur in a subset of GFP^+^ HC-04 cells.

### Primary human hepatocytes support *P*. *falciparum* 3D7HT-GFP EEFs *in vitro*

**Fig 5:**
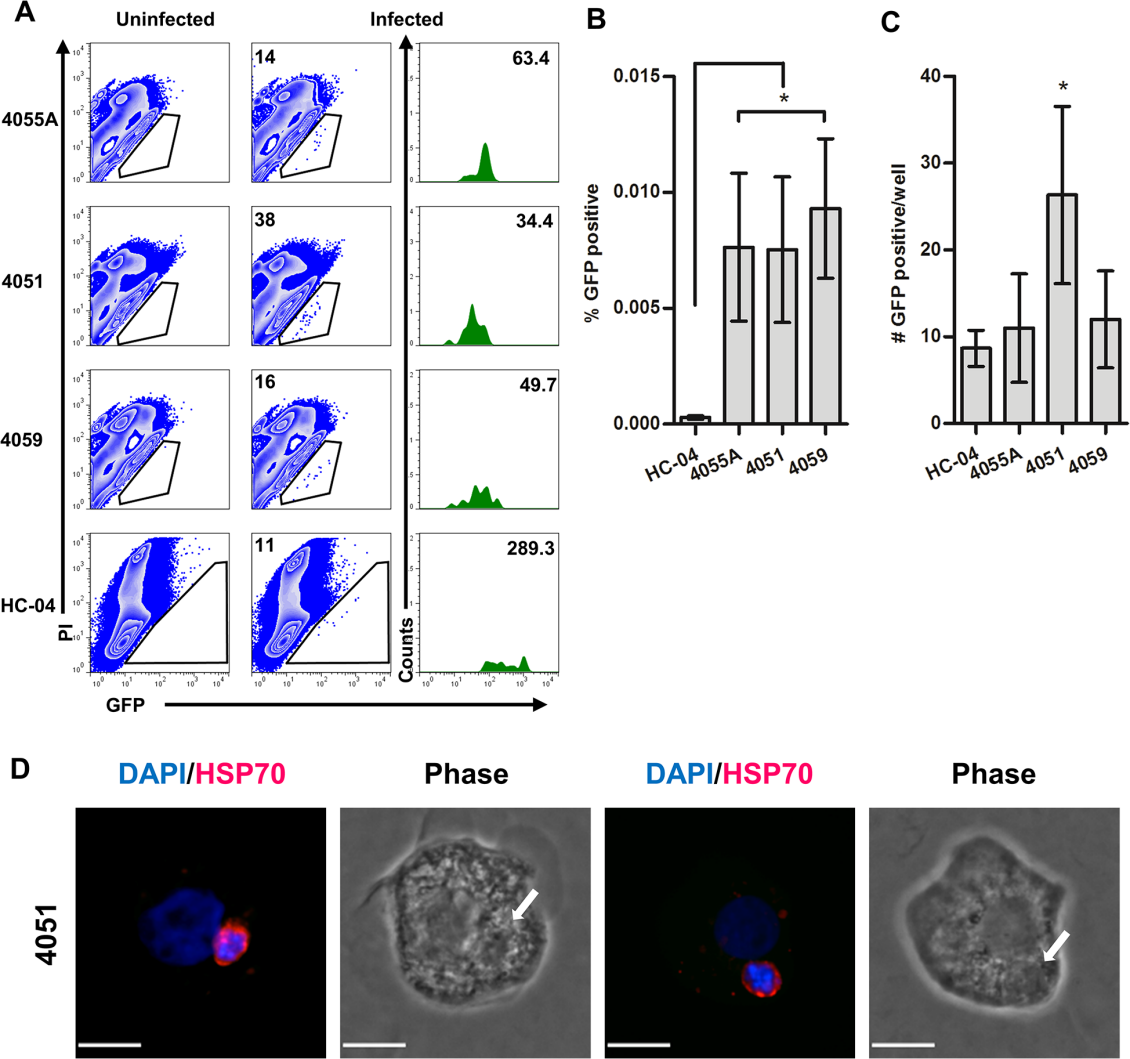
Comparison of detection of *P*. *falciparum* infection in primary human hepatocytes and HC-04. Sporozoites were added at a 1:1 sporozoite-to-hepatocyte ratio. Infected cells were identified by flow cytometry at 96 hours postinfection as GFP-positive events in PI-negative (viable) cell populations (n = 3). Uninfected cultures were used to define the positive gates. (A) Representative gating for detection of GFP events using three human hepatocyte donors and HC-04. Number of GFP-positive PI-negative events indicated (middle panels). Geometric mean of GFP-positive PI-negative events indicated (right panels). (B) Comparisons of the percentage of GFP-positive events and (C) total number of GFP events obtained per well. Mean +/- SD shown. (D) Representative examples of GFP-positive PI-negative events isolated by flow cytometry-based cell sorting 96 hours postinfection and cytospun (Scale bars indicate 10 μm).

We next asked if *P*. *falciparum* infection of HC-04 is comparable to that of primary human hepatocytes. Infected hepatocyte cultures from three donors had viable infected (GFP^+^PI^-^) events detectable by flow cytometry at 96 hours pi (Fig 5A). Higher levels of autofluorescence observed in primary hepatocyte cultures required adjustments of flow cytometer voltages prior to acquisition of primary versus HC-04 cells (S2 Fig). Consequently, direct comparisons of GFP mean fluorescence intensity between HC-04 and primary hepatocyte cultures are not feasible. Unlike HC-04, GFP^+^ events detected in primary hepatocyte cultures were within the range of background autofluorescence in the FL1 fluorescence channel (530/30) (Fig 5A). This highlights the necessity of using a ratio of closely related fluorescent emission channels to distinguish GFP^+^ events from the autofluorescence of primary cells. The relative frequencies of GFP^+^ events detected in primary hepatocytes at 96 hours pi were 20-fold higher (Fig 5A and 5B) than HC-04. However, the numbers of GFP^+^PI^-^ cells in each individual culture (Fig 5C) did not significantly differ between HC-04 and two of the primary hepatocyte cultures (donors 4055A and 4059). Infected hepatocyte cultures from donor 4051 supported 3-fold more GFP^+^ cells than HC-04 and primary cells from the two other donors. The drastic difference in percentages of infected cells found in HC-04 versus primary hepatocyte cultures at 96 hours pi (Fig 5A and 5B) was due to extensive proliferation of uninfected HC-04 cells (Fig 3C). To confirm that GFP^+^ primary hepatocytes contained parasites we sorted and stained cells with an Hsp70-specific antibody (Fig 5D).

**Fig 6:**
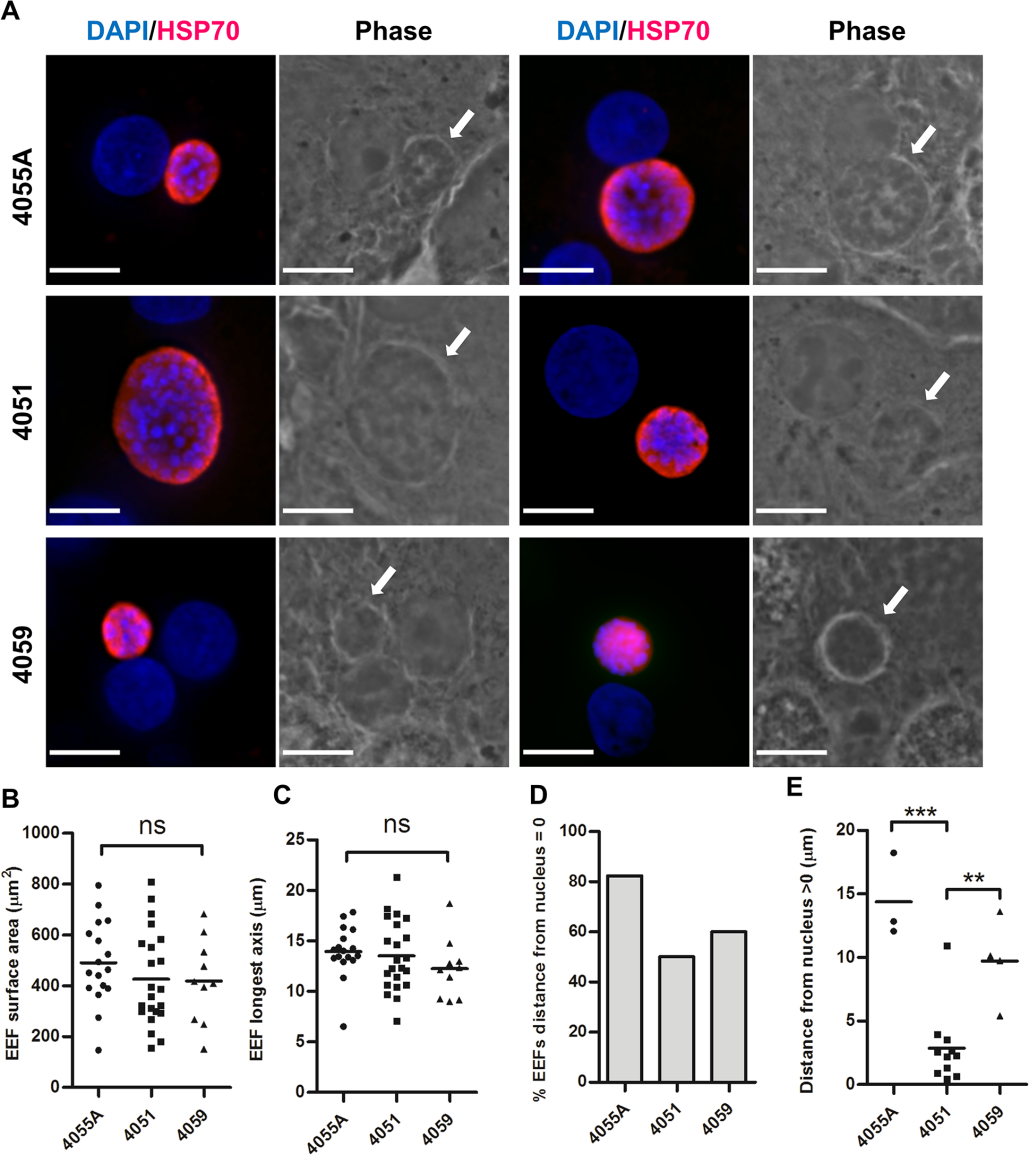
Parasite morphology and size in cryopreserved human hepatocytes from three donors. Sporozoites were added at a 1:1 sporozoite-to-hepatocyte ratio. Cultures were fixed and stained directly on coverslips 96 hours postinfection. (A) Representative micrographs of EEFs observed from each donor. DAPI (blue) represents host and *Plasmodium* nuclei and *Pf*Hsp70 (red) denotes EEF. Arrows indicate EEF in phase contrast micrographs (Scale bar = 10 μM). EEF size was measured by (B) surface area and (C) maximum diameter. (D) Proportion of EEFs observed to be directly adjacent to host nucleus. (E) Of EEFs distant from host nucleus, the distance to the closest nucleus is indicated. Mean shown (horizontal line) for individual dot plots.

The lack of proliferation of primary hepatocytes allowed for visualization of unsorted EEFs maintained on coverslips throughout infection (Fig 6A). We defined parasites by Hsp70 staining (red) and observed no significant differences in parasite size between hepatocytes from different donors at 96 hours pi (Fig 6B and 6C). Similar to previous studies [12, 13], *Pf*3D7-HTGFP parasites were either adjacent to or touching the host nucleus (Fig 6D). A distance of >10μm was observed between some parasites and the closest host nucleus (Fig 6E).

To further substantiate that GFP^+^ cell populations identified in hepatocyte cultures infected with 3D7HT-GFP are specific to infection with sporozoites, we demonstrated that the percentages of GFP^+^ cells in both HC-04 and primary hepatocyte cultures significantly drop if sporozoites are pre-incubated with cytochalasin D prior to infection. Also, a clear dependence of the GFP^+^ cell population size on the sporozoite-to-hepatocyte ratio was observed (S3 Fig). These data are in line with our ability demonstrate that sorted GFP^+^ cell populations (for gating strategy refer to S4 Fig) are composed of cells containing parasites (Figs 4 and 5).

### Dynamics of *Pf*EEF detection by flow cytometry differ in HC-04 and primary hepatocytes

**Fig 7:**
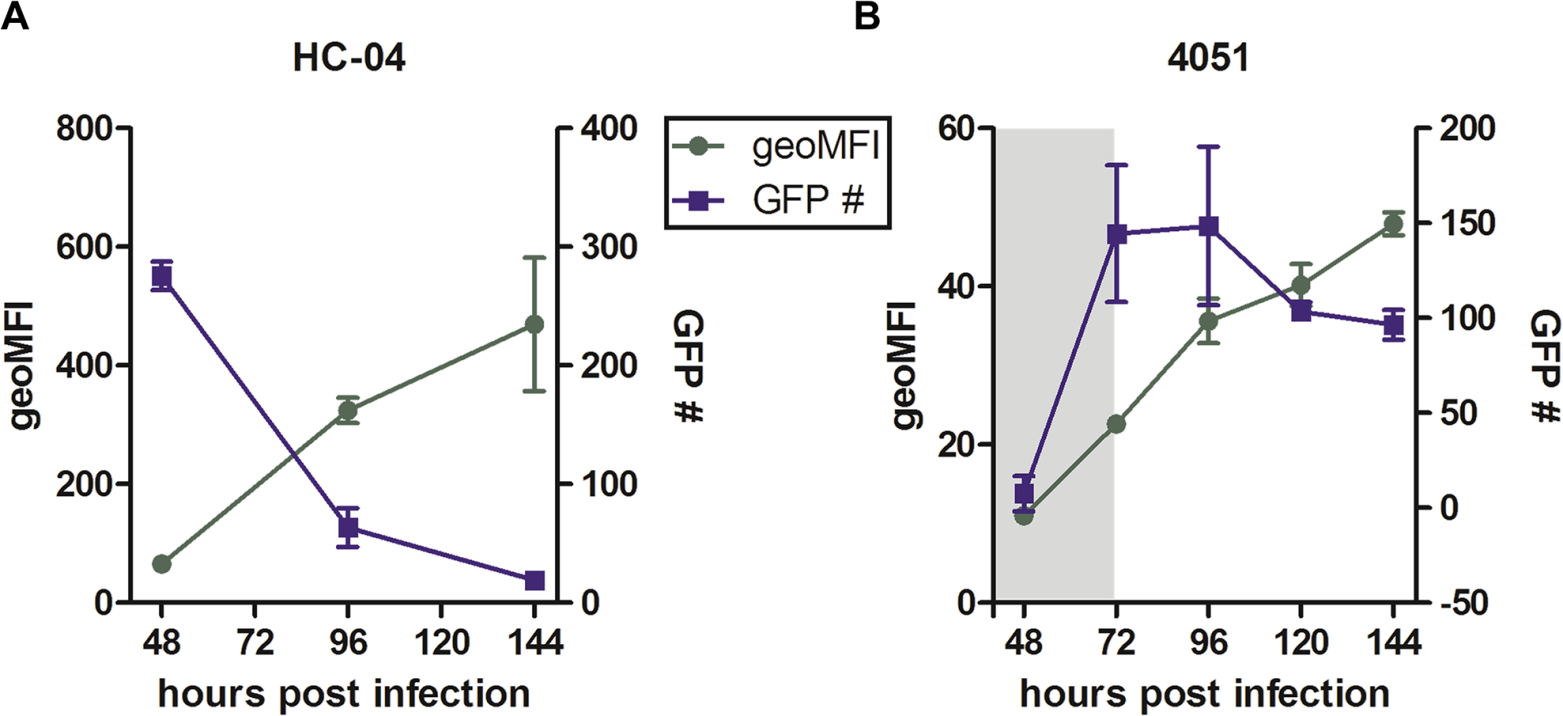
Parasite detection and persistence over time. Sporozoites were added at 0.5:1 sporozoite-to-hepatocyte ratio. Cultures were infected and collected for flow cytometry starting at 48 hours postinfection in duplicate. Plots are shown for the number of GFP-positive events acquired at each time point and the geometric MFI of these events for (A) HC-04 and (B) primary donor 4051 (shading indicates time when a portion of the GFP population is obscured). Mean +/- SD shown.

Our prior experiments were performed at 96 pi to allow for detection of developing parasites. However, the overall ability to detect GFP^+^ parasites by flow cytometry is a function of their brightness over background and attrition over time. To determine the progression of *Pf*EEF development as well as the sensitivity of flow cytometry-based detection of *Pf*3D7HT-GFP EEFs at different time points post infection, we monitored two parameters: the number of viable infected (GFP^+^PI^-^) cells and the intensity of GFP fluorescence in these cells as a marker of parasite development (Fig 7 and S5 Fig).

The maximal numbers of parasites developing in HC-04 cells were detectable starting at 48 hours pi followed by a steady decrease in the number of GFP^+^ events per culture over time (Fig 7A). In contrast, at 48 hours pi detection of parasites in primary hepatocytes was partially obscured by background autofluorescence, even when using the ratio of closely related fluorescence channels. Beginning at 72 hours pi a higher number of parasite-infected cells were detectable in primary hepatocyte cultures by flow cytometry (Fig 7B). The relative parasite survival rate between 96–144 hours pi was 29% for HC-04 and 65% in primary hepatocytes (Fig 7). The parasite-specific GFP fluorescence intensity increased steadily in both HC-04 (Fig 7A) and primary hepatocytes (Fig 7B) and reached its maximum at 144 hours pi, suggesting development of the remaining persisting parasites. Based on these data, for all subsequent experiments we chose to monitor *Pf*3D7HT-GFP development at 48 hours pi in HC-04 and at 96 hours pi in primary hepatocytes, when the maximal numbers of parasite-infected cells were observed in their respective cell cultures.

To determine potential applications of our experimental system, we focused on two major questions relevant to *P*. *falciparum* liver stages: (i) the role of putative host receptors required for efficient infection of human hepatocytes by *P*. *falciparum* parasites and (ii) the role of host factors limiting development of *P*. *falciparum* EEFs.

### CD81 is required for *P*. *falciparum* infection of primary human hepatocytes, but is not essential for the invasion of HC-04

**Fig 8:**
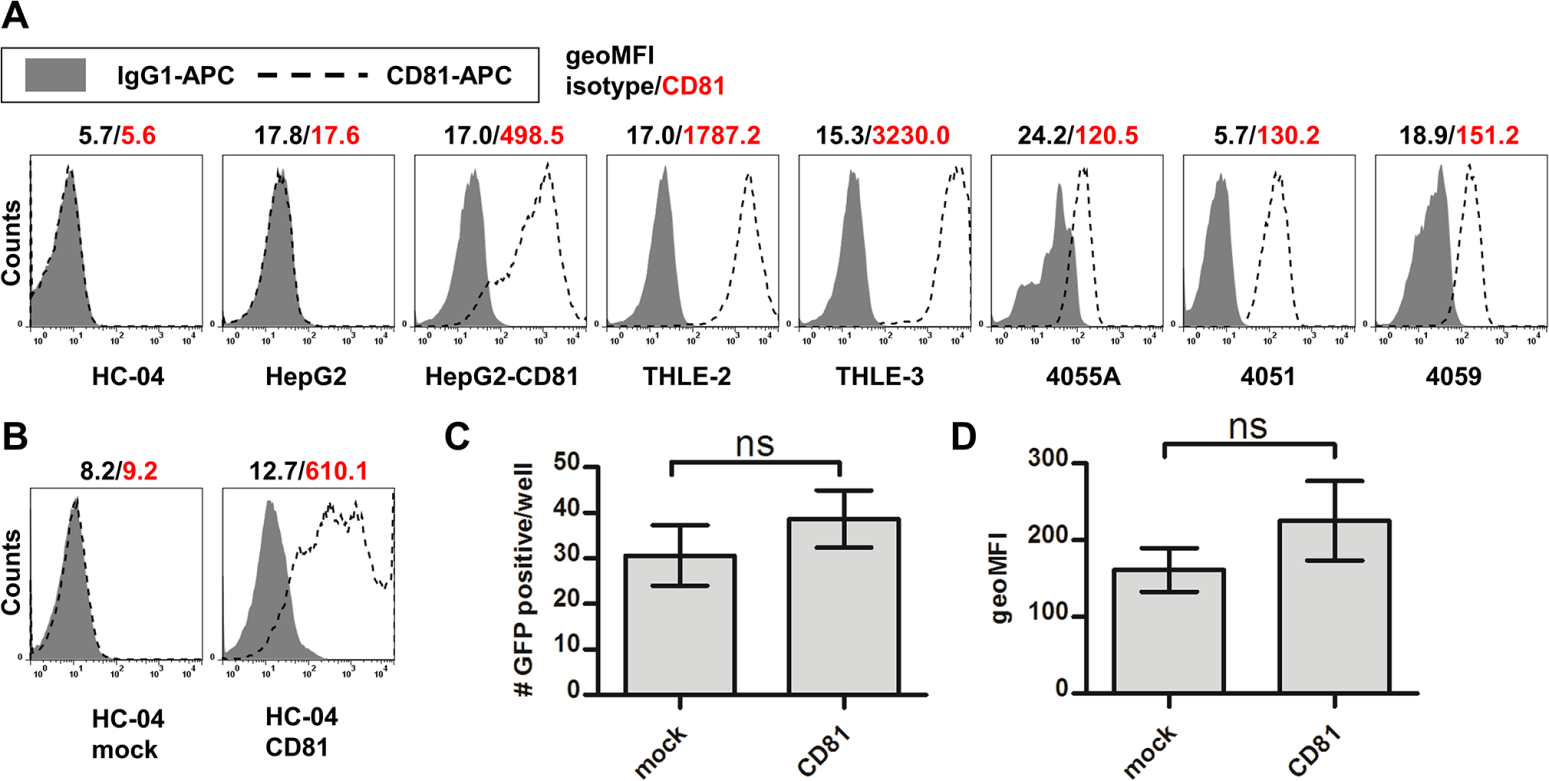
Expression of surface CD81 by human hepatocyte cell lines and primary hepatocyte donors. Surface CD81 was stained using specific antibodies or an isotype control followed by flow cytometry for detection. (A) Representative plots for all cells are shown and geometric MFI is indicated for both isotype (black) and anti-CD81 staining (red). (B) Surface staining of a mock and transient transfection of HC-04. Transiently transfected HC-04 were infected and run on flow cytometry 96 hours postinfection; (C) GFP-positive number and (D) geometric MFI shown. Mean +/- SD shown.

It has been previously shown that functional antibody-based neutralization of CD81 on the surface of primary human hepatocytes abolishes infection by *P*. *falciparum* sporozoites *in vitro* as measured by the numbers of *P*. *falciparum* EEFs detected by immunofluorescence [41]. To address the requirement of CD81 using our experimental model, we first verified the expression of CD81 on the surface of hepatocyte cells lines and primary human hepatocytes. Primary hepatocytes from three donors as well as THLE-2 and -3 cells endogenously expressed surface CD81, whereas neither HC-04 nor HepG2 had detectable expression by flow cytometry (Fig 8A). These data were further confirmed by RT-PCR (data not shown), ruling out our inability to detect low CD81 expression by flow cytometry. These observations in combination with the differential ability of hepatocyte cell lines to support development of *P*. *falciparum* EEFs (see Figs 1–5) reveal no correlation between the expression of CD81 and the susceptibility of human hepatocyte cell lines to *P*. *falciparum* infection or to traversal *in vitro*. Lack of CD81 did not preclude infection of HC-04, and endogenous expression of CD81 on THLE-2 and -3 was not sufficient to allow for efficient infection. Accordingly, ectopic expression of CD81 on HepG2 cells did not result in more efficient *P*. *falciparum* infection (Figs 2 and 3). Similarly, transient expression of CD81 in HC-04 cells did not significantly change the numbers and development of EEFs in these cultures (Fig 8C and 8D).

**Fig 9:**
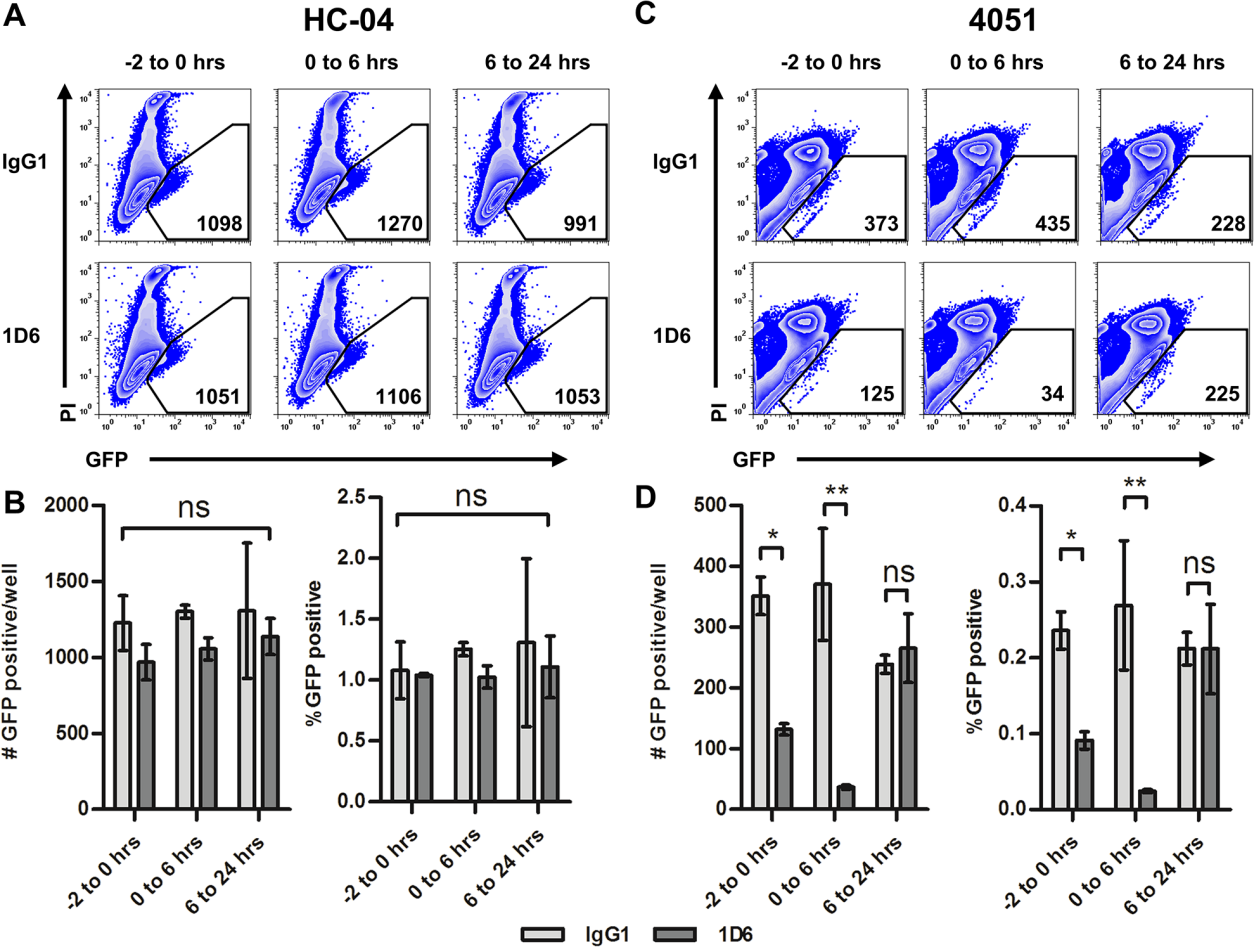
Influence of CD81 blocking by mAb 1D6 on *P*. *falciparum* hepatocyte infection. Sporozoites were added at a 3:1 sporozoite-to-hepatocyte ratio. 1D6 or isotype were added to cultures at 10μg/ml prior to infection (-2 to 0 hours), during invasion (0 to 6 hours) or after invasion (6 to 24 hours). Representative flow plots shown for (A) HC-04 48 hours postinfection and (B) number and percentage of GFP-positive events in duplicate. (C) Flow plots for donor 4051 96 hours postinfection and (D) graphs indicated the number and percentage of GFP-positive events in duplicate. Mean +/- SD shown on all graphs.

Using our experimental model, we recapitulated previously described conditions [41] for antibody-based neutralization of CD81 (Fig 9). We found that the CD81-blocking antibody (clone 1D6) had no effect on *P*. *falciparum* 3D7HT-GFP infection of CD81-negative HC-04, as assessed by percentages and the numbers of infected (GFP^+^PI^-^) cells detected by flow cytometry at 48 hours pi (Fig 9A and 9B). In contrast, the same neutralizing antibody blocked *P*. *falciparum* infection in primary human hepatocytes (Fig 9C and 9D). Moreover, 1D6 did not alter parasite numbers when added 6 hours pi, suggesting that CD81 is critical only during parasite invasion. These data are in agreement with the original observation by Silvie *et al*., demonstrating that CD81 blocking significantly reduces invasion of primary human hepatocytes by *P*. *falciparum* sporozoites [41]. Therefore, though invasion of HC-04 appears to be independent of CD81, we confirm that invasion of primary hepatocytes is CD81-dependent.

### Humanized CSP-specific antibody inhibits traversal and reduces the number of *P*. *falciparum* EEFs in human hepatocytes *in vitro*

**Fig 10:**
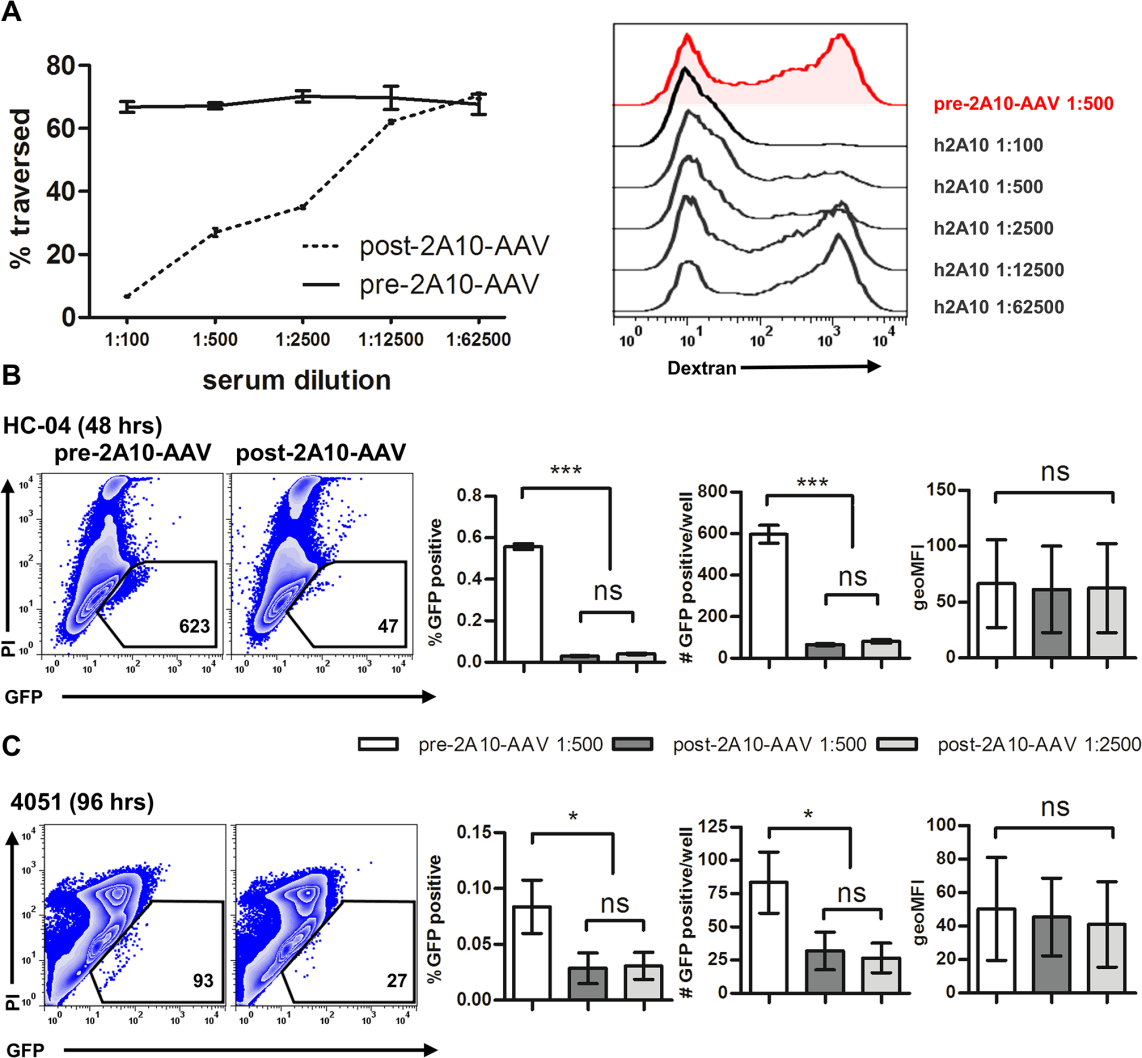
Influence of a humanized anti-CSP mAb 2A10 on *P*. *falciparum* traversal and invasion. Sporozoites were added at a 4.5:1 sporozoite-to-hepatocyte ratio. Cells were infected in the presence of h2A10 or pre-2A10-AAV serum at varying dilutions. (A) Percentage of traversed cells measured by dextran uptake 6 hours postinfection. Representative flow plots for (B) HC-04 48 hours postinfection and (C) donor 4051 96 hours postinfection. Graphs indicated the number, percentage and geometric MFI of GFP-positive events in duplicate. Mean +/- SD shown on all graphs.

The major surface protein of *Plasmodium* sporozoites, the circumsporozoite protein (CSP), is a well-established target for humoral immune responses aimed at neutralization of sporozoite infectivity [42]. To determine the potential utility of our system for the screening of sera for the presence of sporozoite-specific antibodies we characterized the potency of a recently described humanized CSP-specific monoclonal antibody h2A10, produced in a vectored immunoprophylaxis (VIP)-vector transduced mouse [39] (Fig 10). We found that serial dilutions of mouse serum initially containing 1.93 mg/ml of human IgG inhibited parasite traversal in an antibody concentration-dependent manner (Fig 10A). Moreover, h2A10 reduced the relative frequencies and total numbers of GFP^+^PI^-^ cells in both HC-04 and primary hepatocyte cultures (Fig 10B and 10C), although overall inhibition of infection was less prominent in primary cells. Even at a concentration of 3.86 μg/ml (1:500 dilution of h2A10 antibody-containing serum) infection was not completely abolished, suggesting that higher titers of sporozoite antigen-specific antibodies will be required to prevent infection. The intensity of GFP fluorescence in hepatocyte cultures infected along with h2A10 was not significantly different from cultures infected with control sera (Fig 10B and 10C). This suggests that parasites not inhibited by h2A10 during invasion develop normally in infected hepatocytes.

## Discussion

The liver stage of *Plasmodium falciparum* infection, a leading target for generation of protective vaccines, still remains the least characterized part of the parasite lifecycle. Since the demonstration of *P*. *falciparum* EEF development in primary human hepatocytes, a number of alternative methodologies relying on microscopy have been identified to study *P*. *falciparum* liver stages *in vitro* [9, 13]. The generation of fluorescent rodent parasite strains along with a wide range of host cell tropisms has allowed for routine identification and sorting of *Plasmodium* EEFs [40], though such an approach has not been described for *P*. *falciparum*. Using a recently developed strain of *P*. *falciparum* expressing GFP [32] we show that live *P*. *falciparum*-infected hepatocytes are identifiable by flow cytometry and, therefore, can be quantitatively assessed and isolated. This approach allows for sensitive and rapid screening of hepatocyte cell lines and primary human hepatocytes from different donors for their ability to support *Pf*EEF development *in vitro*. Finally, we propose that this approach can be used to quantify the effects of antibodies, cytokines or drugs on parasite invasion and development *in vitro*.

Prior to EEF formation in the liver, *Plasmodium* sporozoites traverse through several host cell types including hepatocytes [23, 26, 43, 44]. We found that *in vitro*, the amount of cells retaining dextran after parasite traversal differed depending on the hepatocyte cell line used (Fig 1). No correlation was observed between the numbers of traversed cells detected 6 hours pi and the frequencies of infected cells detected 96 hours pi (Fig 2). These data suggest that in the cell lines tested the process of invasion and development is distinct from traversal. CSP is shed from the sporozoite surface and is found in both cells permissive or non-permissive to EEF formation [45]. We recently found that flow cytometry-based detection of CSP in *P*. *falciparum*-infected hepatocyte cultures does not represent an accurate measure of hepatocyte infection, but rather reflects the retention of CSP shed during parasite traversal (Trop S. *et al*., submitted). Therefore, we used parasite-encoded GFP as a marker for specific identification of developing parasites by flow cytometry. Using a ratio of two fluorescent channels, a FL1 (530/30) for detection of parasite GFP and FL2 (585/42) for detection of propidium iodide (PI) positive cells, we compared five cell lines (Fig 2) and three primary hepatocyte cultures (Fig 5) for their ability to support *Pf*EEF formation. This approach allows discrimination between the dead/PI-positive (FL1<FL2), PI-negative uninfected/GFP-negative (FL1 = FL2) and GFP-positive (FL1>FL2) cells in infected hepatocyte cultures. This tactic was previously used by others [46] and allows for the exclusion of autofluorescent “false positive” events while detecting weak GFP signals that otherwise would be considered “false negative” if only a single parameter was used.

Among all cell lines tested, HC-04 [28] supported the highest absolute number of *Pf*EEFs at 96 hours pi (Fig 2A and 2C). Surprisingly the HepG2 cell line, previously described as being non-permissive for *P*. *falciparum* sporozoite infection [9], had GFP^+^ events detectable in the viable hepatocyte gate (Fig 2A). The SV40-immortalized hepatocyte lines THLE-2 and THLE-3 [30] had previously not been tested for their ability to support *P*. *falciparum* infection. Though these lines had a low proliferation rate, making them suitable to study the late stages of *Pf*EEFs, both THLE-2 and THLE-3 showed a minimal ability to support *Pf* liver stages in *vitro* (Fig 2A and 2C). In addition to the numbers of GFP^+^ events detectable in infected hepatocyte cultures, we compared the fluorescent intensity of these events as a measure of parasite development in different cell lines. The geometric mean fluorescence intensity (geoMFI) values were assessed in gated GFP^+^ cell populations detected in infected cultures and derived from each well separately. To further verify the presence of developing *P*. *falciparum* EEFs we used quantitative real-time PCR for detection of *Pf*18S at two time points postinfection (Fig 3). Of the five cell lines tested, a time-dependent increase in *Pf*18S gene copy number per each individual well was detected only in HC-04 cultures (Fig 3B), indicating that *P*. *falciparum* parasites can develop in these cells.

Primary human hepatocytes remain the gold standard for generation of *Pf*EEFs *in vitro*. However, recent work has questioned the susceptibility of all primary hepatocytes to *P*. *falciparum* infection, at least *in vitro* [13]. Using flow cytometry we demonstrated, that cryopreserved, commercially available human hepatocytes obtained from 3 different donors were permissive for infection with *P*. *falciparum* 3D7HT-GFP sporozoites. Though efficient detection of parasite-specific GFP signal at the early stages of infection was hampered due to high autofluorescence in normal hepatocytes [9, 47, 48], primary cells infected with *Pf*3D7HT-GFP were reproducibly detectable by flow cytometry between 72–144 hours pi (Fig 7B). It needs to be seen if flow cytometry based detection can be applied to the *ex vivo* detection of the liver stage parasites in the recently developed humanized mouse models of *P*. *falciparum* infection [16].

To address the dynamics of 3D7HT-GFP parasite detection and development in hepatocytes *in vitro* we used flow cytometry to monitor changes in parasite numbers and parasite-specific GFP fluorescence (MFI) as a reflection of parasite expansion within infected hepatocytes. The maximum numbers of GFP^+^ events in HC-04 cultures were detected at 48 hours pi followed by a rapid decline (Fig 7A). In contrast, a larger proportion of infected cells persisted from 96 to 144 hours pi in primary hepatocytes (Fig 7B). A similar trend has been observed by others [13]. Importantly, an increase in GFP fluorescence over time was seen in infected cells that persisted in both HC-04 and primary hepatocyte cultures, indicative of parasite development. Taken together, these results show that *P*. *falciparum* parasites can develop in both HC-04 and primary hepatocytes.

To visualize the presence of parasites in GFP^+^ cells and to demonstrate the ability to selectively isolate live parasites from bulk infected cultures we sorted GFP^+^ HC-04 cells and primary hepatocytes (Figs 4 and 5). It does not appear that *Pf*EEFs developing in HC-04 cells exclusively localize to the host nucleus, a phenomenon that has been previously observed in HepG2 cells [29]. Using GFP and *Pf*Hsp70 as parasite-specific markers, we identified three patterns of *P*. *falciparum* parasites in HC-04 cells at 96 hours pi. Parasites were found either adjacent to an intact host nucleus, surrounded by a fragmenting or dividing host nuclei or without an apparent host nucleus distinct from the parasite DNA (Fig 4). Additional studies are needed to describe phenotypes of parasites detected in HC-04 cells at the later time points of infection. In contrast to parasites developing in HC-04, *Pf*EEFs detected in sorted GFP^+^ primary hepatocytes were mainly adjacent to the intact host nucleus easily distinguishable from the parasite nuclei (Fig 5D).

In contrast to proliferating HC-04 cells that rapidly form three-dimensional cell cultures composed of uninfected cells overlaying parasite-infected cells, non-proliferating primary hepatocytes permit identification of EEFs by fluorescent microscopy without prior sorting (Fig 6). Unlike sorting and cytospin procedures, direct fixation of infected cells on coverslips better preserves both parasite and host hepatocyte morphology. In all three primary infected hepatocyte cultures, *Pf*Hsp70-specific antibody identified developing parasites (Fig 6), but GFP fluorescence was not detectable in these cells by fluorescence microscopy (data not shown). As discussed above, the inability of GFP signal to be detected by fluorescence microscopy is likely due to high autofluorescence in normal hepatocytes [9] (S2 Fig). At 96 hours pi *Pf*EEFs developing in primary hepatocyte cultures were mainly adjacent to the host nucleus and comparable in size to parasites described *in vivo* [16] (Fig 6).

After establishing a model of *in vitro Pf*EEF detection and verifying the presence of parasites in both primary hepatocyte and HC-04 cell cultures, we designed several experiments to explore potential applications of our model for both basic and translational studies. Ligands on the human hepatocyte cell surface facilitating invasion by *P*. *falciparum* sporozoites are still poorly understood. We re-examined the necessity of CD81 expression for invasion by *P*. *falciparum* sporozoites. Previous studies determined that blocking of CD81 during *P*. *falciparum* infection reduces EEF numbers in primary hepatocytes [41], however expression of CD81 on non-permissive cell types was insufficient to confer invasion [29]. Here we show that endogenous surface expression of CD81 (Fig 8A) did not correlate with *P*. *falciparum* EEF numbers in the five cell lines tested (Fig 2). Primary hepatocytes derived from 3 human donors were permissive for *P*. *falciparum* infection and had expression of CD81 on the cell surface (Fig 8A). HC-04, the most permissive to *P*. *falciparum* infection among all cell lines tested, lacks CD81 and transient ectopic expression of CD81 did not alter susceptibility of HC-04 cells to invasion (Fig 8C and 8D). Taking advantage of the ability to quantitatively assess EEF development by flow cytometry, we re-examined the requirement for CD81 expression during *P*. *falciparum* sporozoite invasion (Fig 9). We found that the CD81-blocking monoclonal antibody 1D6 did not alter *P*. *falciparum* infection of HC-04 cells (Fig 9A and 9B). However, in agreement with the original finding by Silvie *et al*. [41], the same antibody decreased the *Pf*EEF numbers in primary human hepatocyte cultures when used during sporozoite invasion (Fig 9C and 9D). The inability of 1D6 to alter sporozoite invasion of CD81-negative HC-04 suggests that 1D6 has no unspecific effect on sporozoites. In agreement, pre-incubation of primary hepatocytes with 1D6 followed by its removal prior to infection blocked sporozoite invasion. Additionally, no effect of 1D6 was observed when added six hours after infection in either HC-04 or primary hepatocyte cultures. Our findings suggest that different host cell surface receptors and/or distinct parasite invasion pathways can be utilized by *P*. *falciparum* sporozoites to establish liver stage infection. One possibility is that differences in heparan sulfate proteoglycans (HSPG) between HC-04 and primary hepatocytes mediate a differential requirement for CD81. It has been established that CSP binds to HSPGs and influences sporozoite invasion [49–51] but does so more prominently under dynamic flow conditions [52]. Alternatively, the inability of CD81 antibodies to alter GFP^+^ parasite numbers detected in infected HC-04 cell cultures by flow cytometry could reflect the persistence of transmigrating sporozoites trapped in a proportion of hepatocytes. Novel technologies based on combining flow cytometry and fluorescence microscopy may prove useful in addressing this issue. We propose that our experimental approach can facilitate the discovery of additional human host factors or further characterize the importance of HSPGs during *P*. *falciparum* sporozoite invasion.

Next we demonstrated the utility of these methods to quantify the effects of anti-sporozoite antibodies on traversal and invasion. Sterilizing immunity to the liver stage of *Plasmodium* infection does not occur during natural infection [53]. It has been well established that antibodies directed against the sporozoite are capable of blocking parasite motility *in vitro* and *in vivo* and can consequently reduce parasite invasion into hepatocytes [54–56]. The RTS,S vaccine is capable of inducing anti-CSP antibodies able to protect against *P*. *falciparum* infection in a dose dependent manner [17]. Inhibition of parasite motility as measured by traversal *in vitro* has been used to characterize humoral responses against the sporozoite [57]. However, to establish a correlation between the effects exerted by parasite-specific antibodies on traversal with effects on formation of *Pf*EEFs has required a humanized mouse model [17, 57]. Here, using serum from a vectored immunoprophylaxis (VIP)-vector transduced mouse producing a humanized *Pf*CSP-specific monoclonal antibody [39], we demonstrate an inhibition of both traversal and infection by *P*. *falciparum* sporozoites *in vitro* (Fig 10). Since our approach is not limited to a specific parasite antigen, it may be applied to screening of sera from human cohorts immunized with radiation attenuated (RAS), genetically attenuated (GAS) sporozoites, or sporozoites combined with chloroquine chemoprophylaxis (CPS) based protection [20, 58]. *In vitro* methods enabling objective evaluation of anti-sporozoite humoral immune responses are particularly advantageous because they require considerably less material than humanized mouse models and can include multiple biological replicates. However, it should be stated that *in vitro* models of infection lack the complex hepatic architecture and cannot entirely recapitulate the natural infection.

Previously, detection of *Pf*EEFs by flow cytometry has only been possible using rodent *Plasmodium* species [9, 24, 33, 40]. Although the use of rodent parasites and mouse models of *Plasmodium* infection will continue to be a cornerstone of malaria research, advancements in methods for studying *Pf*EEFs *in vitro* are required to strengthen our understanding of basic parasite biology and to provide objective and quantifiable protocols to inform translational research. HC-04 and primary hepatocytes constitute complementary *in vitro* models to study *Pf*EEF formation. Among five cell lines tested, HC-04 is able to support the greatest number of developing parasites. However, uninfected cells in HC-04 cultures rapidly proliferate, complicating parasite detection after 72–96 hours pi. On the other hand, parasite traversal can be quantified in these cells early after infection and fluorescent parasites are clearly detectable by flow cytometry starting at 48 hours pi. Consequently, HC-04 is a suitable host cell to screen for the efficacy of interventions aimed at blocking the motility of sporozoites, such as anti-parasite antibodies, which can be assessed in traversal assays. However, the HC-04 cell line is not fully suitable for study of late *Pf* liver stages.

In contrast to HC-04 cells, primary hepatocytes do not proliferate *in vitro*, however, the *Pf*EEFs can be clearly detected in these cells by flow cytometry only beginning at 72 hours pi. Though primary hepatocytes will continue to be the standard for study of *Pf*EEFs, *de novo* generation or identification of additional human hepatocyte cell lines with low doubling times and limited autofluorescence still remains a priority. The development of a *P*. *falciparum* line expressing GFP through the parasite’s lifecycle [32] was paramount in our ability to reproducibly detect and quantify *Pf*EEFs by flow cytometry. The generation of a novel *P*. *falciparum* strain expressing a fluorophore brighter than GFP and/or use of a stronger liver stage promoter will facilitate flow cytometry-based detection and isolation of *P*. *falciparum* parasites in human hepatocytes even before 48 hours postinfection.

## Supporting Information

S1 Fig Specificity of detection of traversal of HC-04 cells by *P. falciparum* sporozoites. (A) Gating strategy used to detect cells traversed by sporozoites 6 hours after infection. (B) Traversal is inhibited when motility of sporozoites is blocked prior to infection with cytochalasin D (10μM, 10 min at RT). Representative dot plots demonstrating effect of cytochalasin D. (C) Percentage of traversed cells depends on sporozoite-to-hepatocyte ratio. Effect of cytochalasin D on cells traversal in hepatocyte cultures incubated with *P*. *falciparum* sporozoites at a range of sporozoite-to-hepatocyte ratio.
(TIF)

S2 Fig Differences in background autofluorescence between primary hepatocytes and the HC-04 cell line necessitated adjusted flow cytometer voltage settings during acquisition. HC-04 and primary hepatocytes have different basal autofluorescence characteristics. Two different voltage settings (designated as “high” and “low”) used for acquisition are indicated in the table and demonstrate the high intrinsic autofluorescence seen in primary human hepatocytes.
(TIF)

S3 Fig Specificity of detection of infection by *P. falciparum* sporozoites. The percentage of GFP^+^ cells depends on sporozoite-to-hepatocyte ratio. Preincubation of sporozoites with cytochalasin D prior to infection (10μM, 10 min at RT) reduces the percentages of GFP^+^ cells detected by flow cytometry (A) 48 hours after infection in HC-04 and (B) 96 hours after infection in primary human hepatocytes. (C) Representative plots demonstrate the effect of cytochalasin D on the number of GFP positive cells detected in HC-04 and primary hepatocyte cultures.
(TIF)

S4 Fig Gating strategy for specific isolation of GFP+ hepatocytes infected with *P. falciparum* 3D7HT-GFP sporozoites. Gating strategy prior to sorting is shown for (A) HC-04 cells and (B) primary human hepatocytes. Initial gating on forward and side scatter characteristics followed doublet exclusion by pulse width and identification of PI-negative GFP-positive cells using a FL1/FL2 ratio. Data shown are from 10^7^ events acquired.
(TIF)

S5 Fig Detection of GFP+ cells in *P. falciparum* 3D7HT-GFP-infected cultures in time kinetic. Sporozoites were added at 0.5:1 sporozoite-to-hepatocyte ratio to **(A)** HC-04 and (B) primary hepatocyte cultures. Representative plots are shown.
(TIF)
