# Supplementary figures and images for: Flow Cytometry Based Detection and Isolation of Plasmodium falciparum Liver Stages In Vitro
Source: PLoS One. 2015 Jun 12;10(6):e0129623. doi: 10.1371/journal.pone.0129623 (PMC4466555; doi:10.1371/journal.pone.0129623)

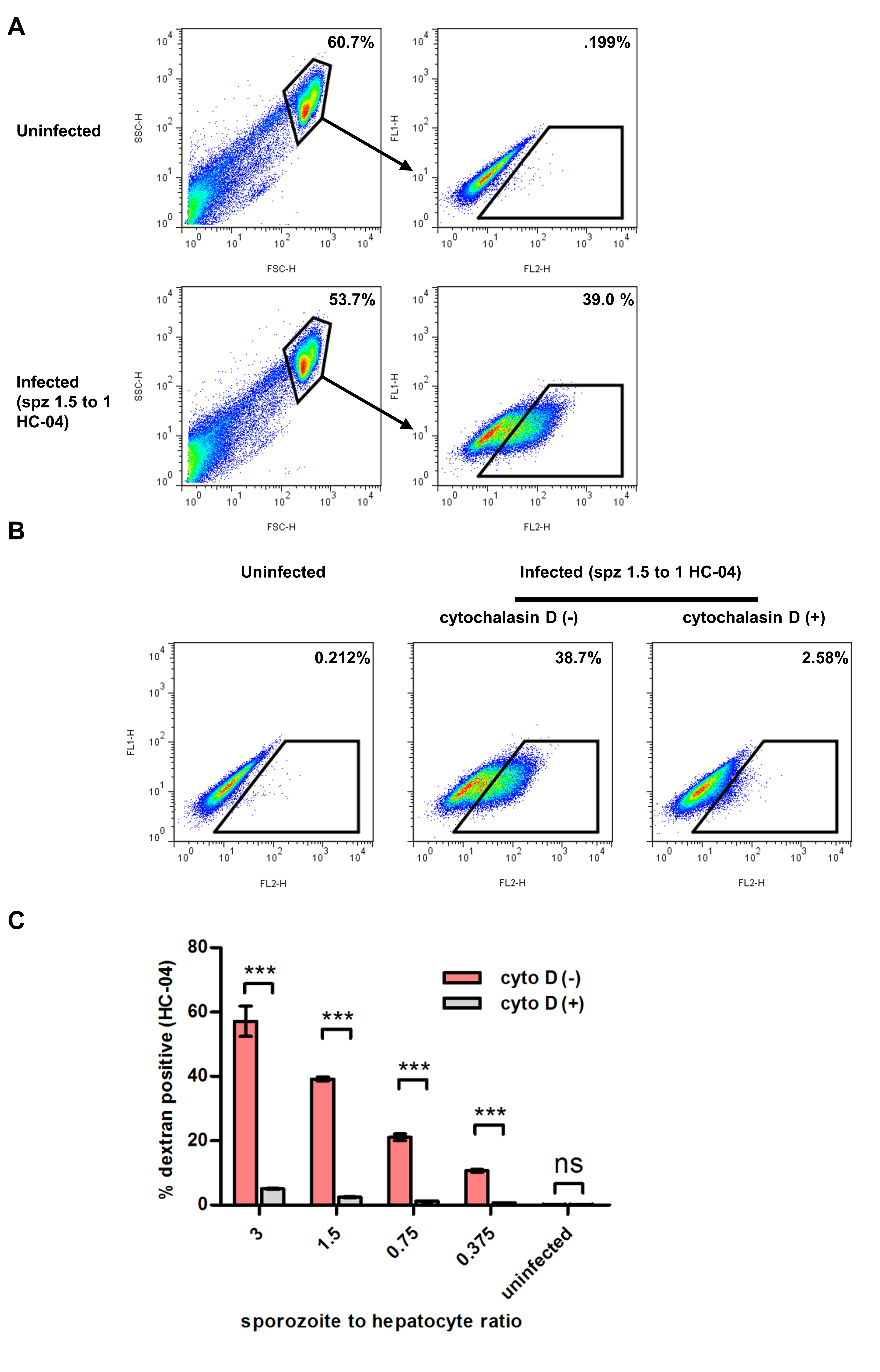

Supplement: S1 Fig — (A) Gating strategy used to detect cells traversed by sporozoites 6 hours after infection. (B) Traversal is inhibited when motility of sporozoites is blocked prior to infection with cytochalasin D (10μM, 10 min at RT). Representative dot plots demonstrating effect of cytochalasin D. (C) Percentage of traversed cells depends on sporozoite-to-hepatocyte ratio. Effect of cytochalasin D on cells traversal in hepatocyte cultures incubated with P. falciparum sporozoites at a range of sporozoite-to-hepatocyte ratio. (TIF) [file pone.0129623.s001.tif]

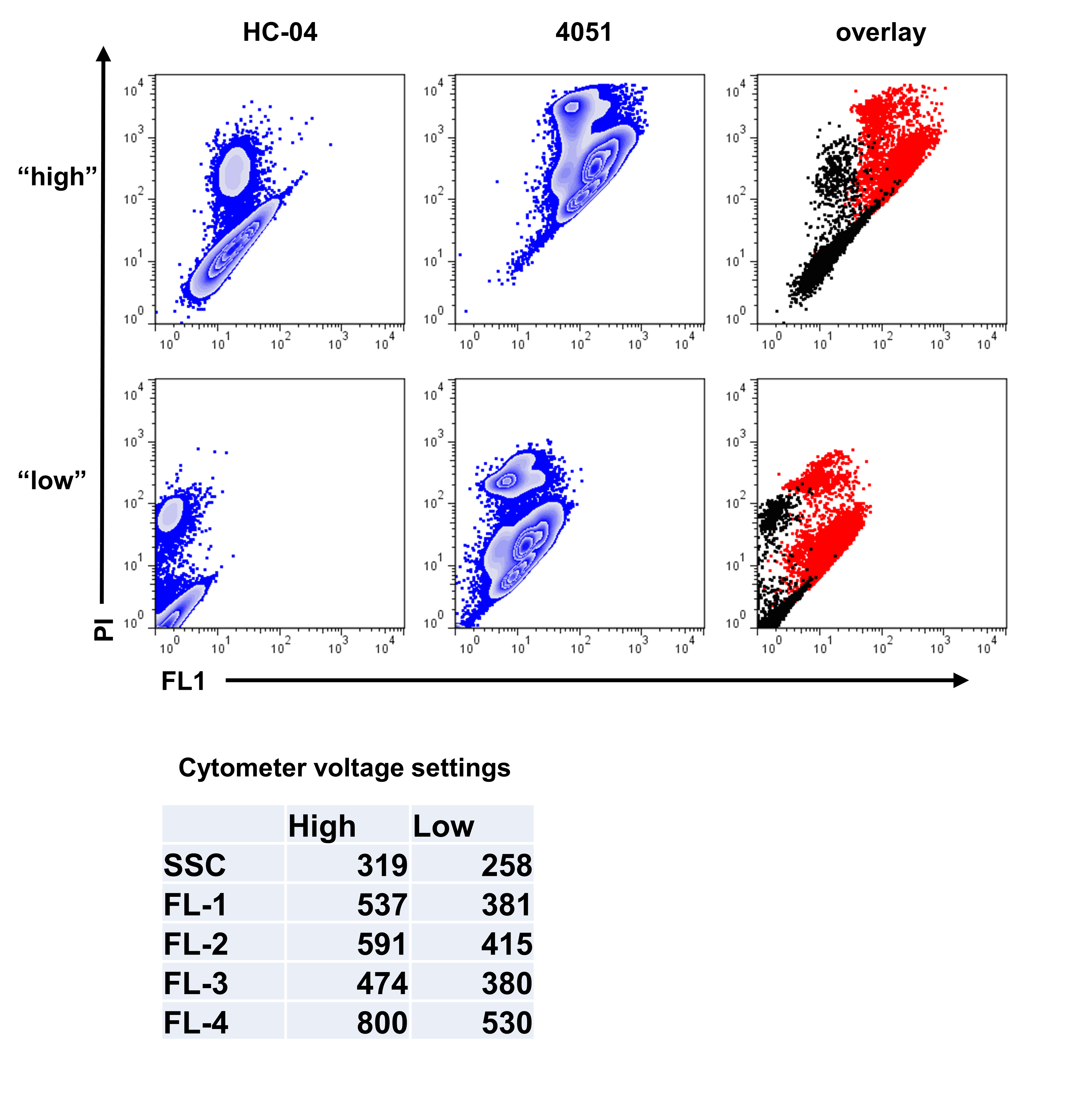

Supplement: S2 Fig — HC-04 and primary hepatocytes have different basal autofluorescence characteristics. Two different voltage settings (designated as “high” and “low”) used for acquisition are indicated in the table and demonstrate the high intrinsic autofluorescence seen in primary human hepatocytes. (TIF) [file pone.0129623.s002.tif]

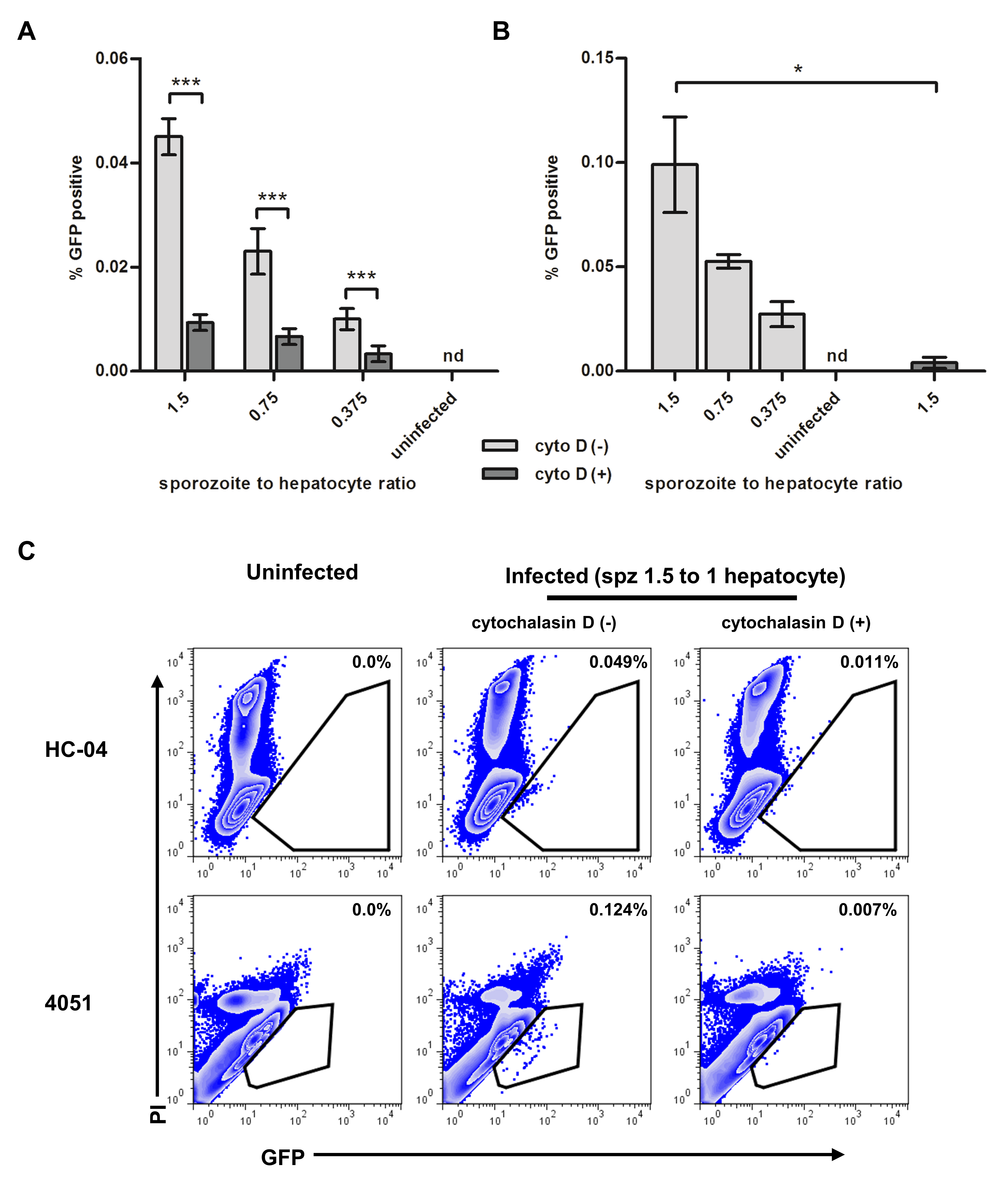

Supplement: S3 Fig — The percentage of GFP+ cells depends on sporozoite-to-hepatocyte ratio. Preincubation of sporozoites with cytochalasin D prior to infection (10μM, 10 min at RT) reduces the percentages of GFP+ cells detected by flow cytometry (A) 48 hours after infection in HC-04 and (B) 96 hours after infection in primary human hepatocytes. (C) Representative plots demonstrate the effect of cytochalasin D on the number of GFP positive cells detected in HC-04 and primary hepatocyte cultures. (TIF) [file pone.0129623.s003.tif]

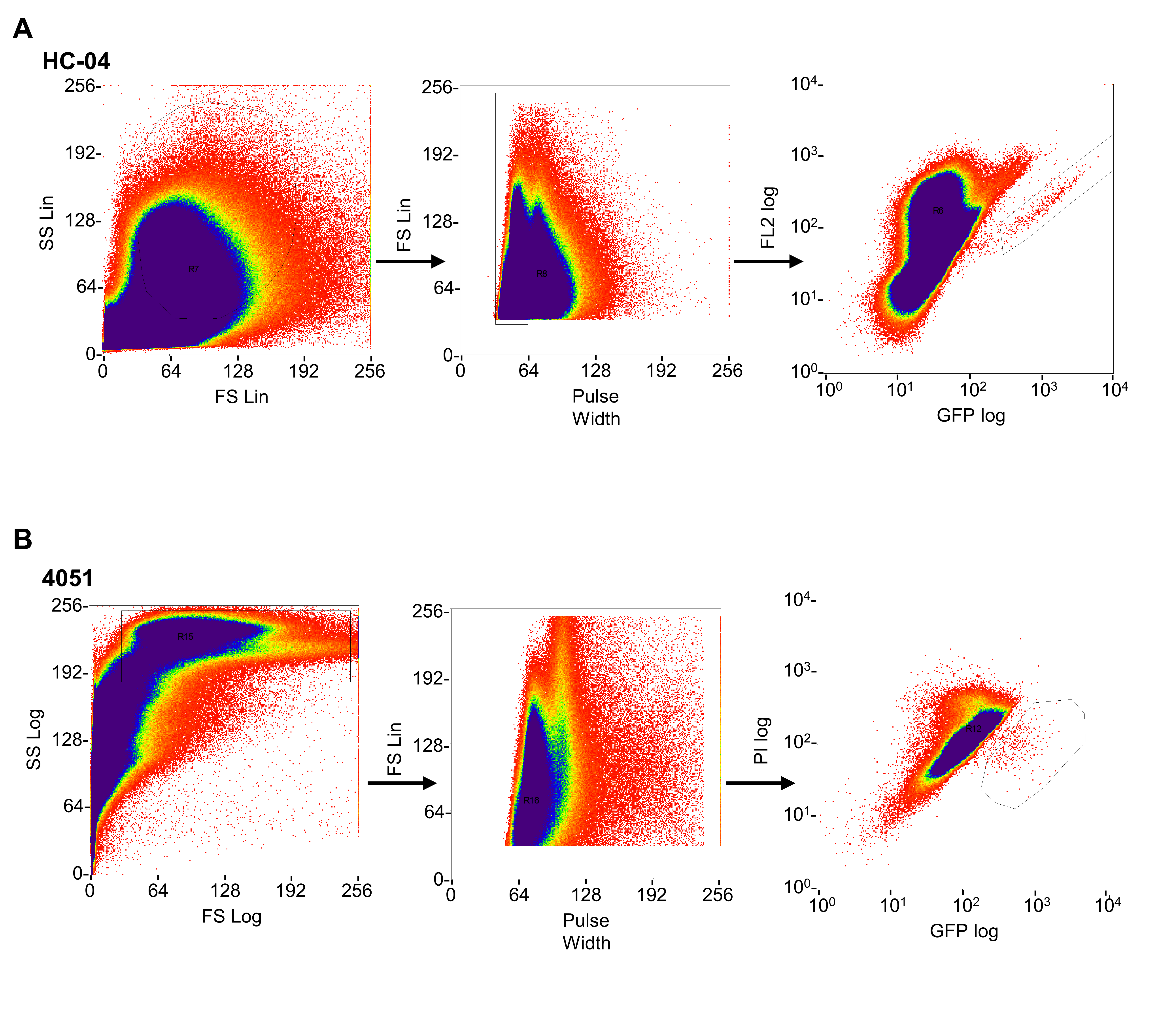

Supplement: S4 Fig — Gating strategy prior to sorting is shown for (A) HC-04 cells and (B) primary human hepatocytes. Initial gating on forward and side scatter characteristics followed doublet exclusion by pulse width and identification of PI-negative GFP-positive cells using a FL1/FL2 ratio. Data shown are from 107 events acquired. (TIF) [file pone.0129623.s004.tif]

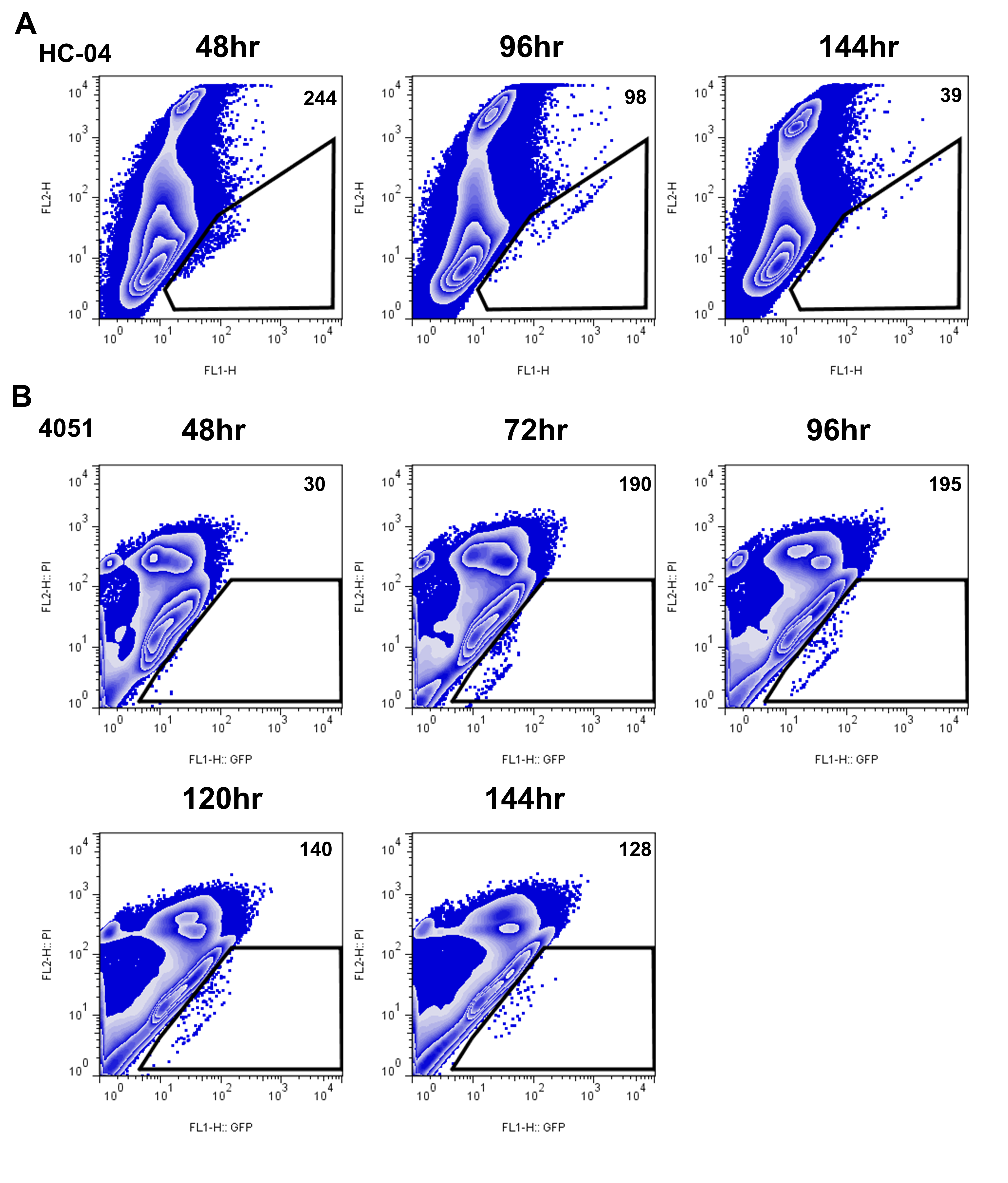

Supplement: S5 Fig — Sporozoites were added at 0.5:1 sporozoite-to-hepatocyte ratio to (A) HC-04 and (B) primary hepatocyte cultures. Representative plots are shown. (TIF) [file pone.0129623.s005.tif]
